# SDePER: a hybrid machine learning and regression method for cell-type deconvolution of spatial barcoding-based transcriptomic data

**DOI:** 10.1186/s13059-024-03416-2

**Published:** 2024-10-14

**Authors:** Yunqing Liu, Ningshan Li, Ji Qi, Gang Xu, Jiayi Zhao, Nating Wang, Xiayuan Huang, Wenhao Jiang, Huanhuan Wei, Aurélien Justet, Taylor S. Adams, Robert Homer, Amei Amei, Ivan O. Rosas, Naftali Kaminski, Zuoheng Wang, Xiting Yan

**Affiliations:** 1 Department of Biostatistics, Yale School of Public Health, New Haven, CT USA; 2 SJTU-Yale Join Center for Biostatistics and Data Science, Department of Bioinformatics and Biostatistics, School of Life Sciences and Biotechnology, Shanghai Jiao Tong University, Shanghai, China; 3 The Second Affiliated Hospital of The Chinese University of Hong Kong, Shenzhen, Shenzhen, Guangdong China; 4 Department of Mathematical Sciences, University of Nevada, Las Vegas, NV USA; 5 Section of Pulmonary, Critical Care and Sleep Medicine, Yale School of Medicine, New Haven, CT USA; 6 Service de Pneumologie, Centre de Competences de Maladies Pulmonaires Rares, CHU de Caen UNICAEN, CEA, CNRS, ISTCT/CERVOxy Group, GIP CYCERON, Normandie University, Caen, France; 7 Department of Pathology, Yale School of Medicine, New Haven, CT USA; 8 Department of Medicine, Baylor College of Medicine, Houston, TX USA; 9 Department of Biomedical Informatics & Data Science, Yale School of Medicine, New Haven, CT USA

## Abstract

Spatial barcoding-based transcriptomic (ST) data require deconvolution for cellular-level downstream analysis. Here we present SDePER, a hybrid machine learning and regression method to deconvolve ST data using reference single-cell RNA sequencing (scRNA-seq) data. SDePER tackles platform effects between ST and scRNA-seq data, ensuring a linear relationship between them while addressing sparsity and spatial correlations in cell types across capture spots. SDePER estimates cell-type proportions, enabling enhanced resolution tissue mapping by imputing cell-type compositions and gene expressions at unmeasured locations. Applications to simulated data and four real datasets showed SDePER’s superior accuracy and robustness over existing methods.

**Supplementary Information:**

The online version contains supplementary material available at 10.1186/s13059-024-03416-2.

## Background

Spatial transcriptomic technologies enabled measuring gene expression and physical locations of spots and/or cells simultaneously in intact tissues of various types in an unbiased and high-throughput way [1–4], providing unprecedented information to understand disease-associated changes. Specifically, the spatial barcoding-based (ST) technologies, such as Slide-seq [5], HDST [6], ST [4], and 10 × Genomics Visium, divide tissue into small capture spots and measure high-throughput gene expression levels unbiasedly for each spot with known physical location [4–10]. Depending on the size of capture spots, the measured expression profile is an average expression profile of cells of unknown types. Therefore, the corresponding data lacks single-cell resolution [11] and requires cell-type deconvolution to understand the cell-type composition and cell-type-specific gene expression in each spot.

One common way to deconvolve ST data is to use cell-type-specific expression profile from existing single-cell RNA sequencing (scRNA-seq) data of the same tissue type. Many methods have been developed [12–28], which can be divided into four categories: machine learning-based [20–22], regression-based [23–28], statistical modeling-based [12–15], and data mapping-based methods [16]. Benchmarking studies have been conducted to compare the performance of these methods [29–31].

Despite the success of current methods, the following three challenges have not been well addressed and, more importantly, no method addresses them simultaneously. First, systematic difference exists between scRNA-seq and ST data [12–15, 22, 24, 25] due to various technical factors, such as differences in protocols, reagents, platforms, or simply sequencing depths. This systematic difference, termed as platform effects [12], makes the relationship between ST data and cell-type-specific expression profiles from the reference scRNA-seq data non-linear and varying across different technologies. A few statistical model-based methods [12–15] consider the platform effects as multiplicative random or fixed effect. However, these methods were shown in a previous benchmarking study [29] to have comparable performance to methods that do not address platform effects, leaving it unclear whether platform effects were adequately addressed. DSTG and some of the data mapping-based methods implicitly addressed platform effects by embedding scRNA-seq or scRNA-seq-derived pseudo-spot data and real ST data into a common latent space. Second, among all cell types existed in the tissue, only a few cell types are present in each spot. For example, 38 different cell types were found in the scRNA-seq data of whole lung tissues (the IPF dataset in real data analyses). However, capture spots of the 10 × Genomics Visium platform with a size of ~ 55 μm contained only 2–10 cells per spot, demonstrating a sparse presentation of all cell types existed in the tissue. This sparsity was considered by RCTD, SPOTlight, DestVI, and SpatialDWLS but using subjective hard thresholding. Lastly, previous studies [24, 32, 33] have shown that cell-type composition of spots that are physically close in the tissue tend to be similar or correlated. Only CARD explicitly considered the across-spot spatial correlation of cell-type compositions.

**Fig. 1:**
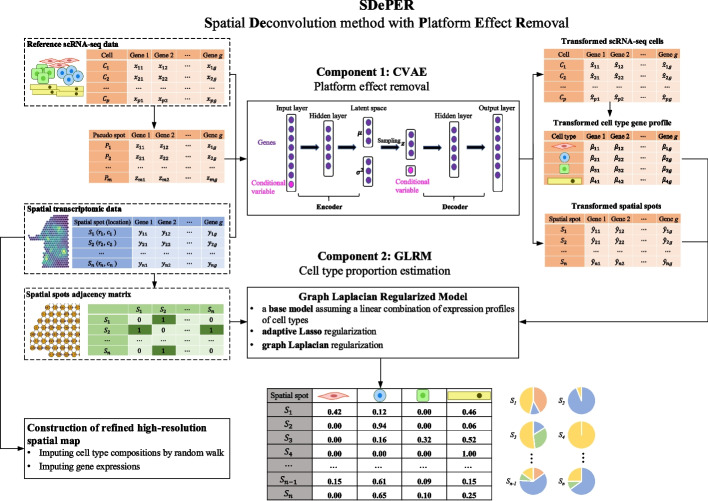
Schematic overview of SDePER. SDePER performs cell-type deconvolution of ST data in a two-step fashion. In the first step, conditional variational autoencoder (CVAE) takes three datasets as input: real ST data, reference scRNA-seq data, and pseudo-spot data generated using the reference scRNA-seq data. Using the trained encoder and decoder under the two conditions (ST and scRNA-seq), real ST data is transformed into the same space as scRNA-seq data and pseudo-spot data. The transformed real ST data and cell type-specific expression profiles are then used to fit the graph Laplacian regularized model (GLRM) with penalties for sparsity and across-spot spatial correction in cell-type compositions. The estimated cell-type compositions from GLRM can be further used to impute for cell-type compositions and gene expression at unmeasured locations in the original spatial map to construct new spatial map at arbitrarily higher resolution

To address all the aforementioned challenges, we propose a two-step hybrid machine learning and regression method, SDePER, that considers platform effects removal, spatial correlation, and sparsity (Fig. 1). In the first step, a conditional variational autoencoder (CVAE) [34] is used to adjust the ST and reference scRNA-seq data for platform effects removal. In the second step, a graph Laplacian regularized model (GLRM) is fitted to the adjusted ST data with consideration of the spatial correlation of cell-type compositions between neighboring spots and sparsity of present cell types per spot. Based on the estimated cell-type compositions, a random walk is performed to impute cell-type compositions and gene expression at unmeasured locations in a tissue map with enhanced resolution. We demonstrate the advantage of SDePER through extensive simulations and applications to four real datasets from various tissues, species, and technologies.

## Results

### SDePER—efficiently corrects for platform effects

**Fig. 2:**
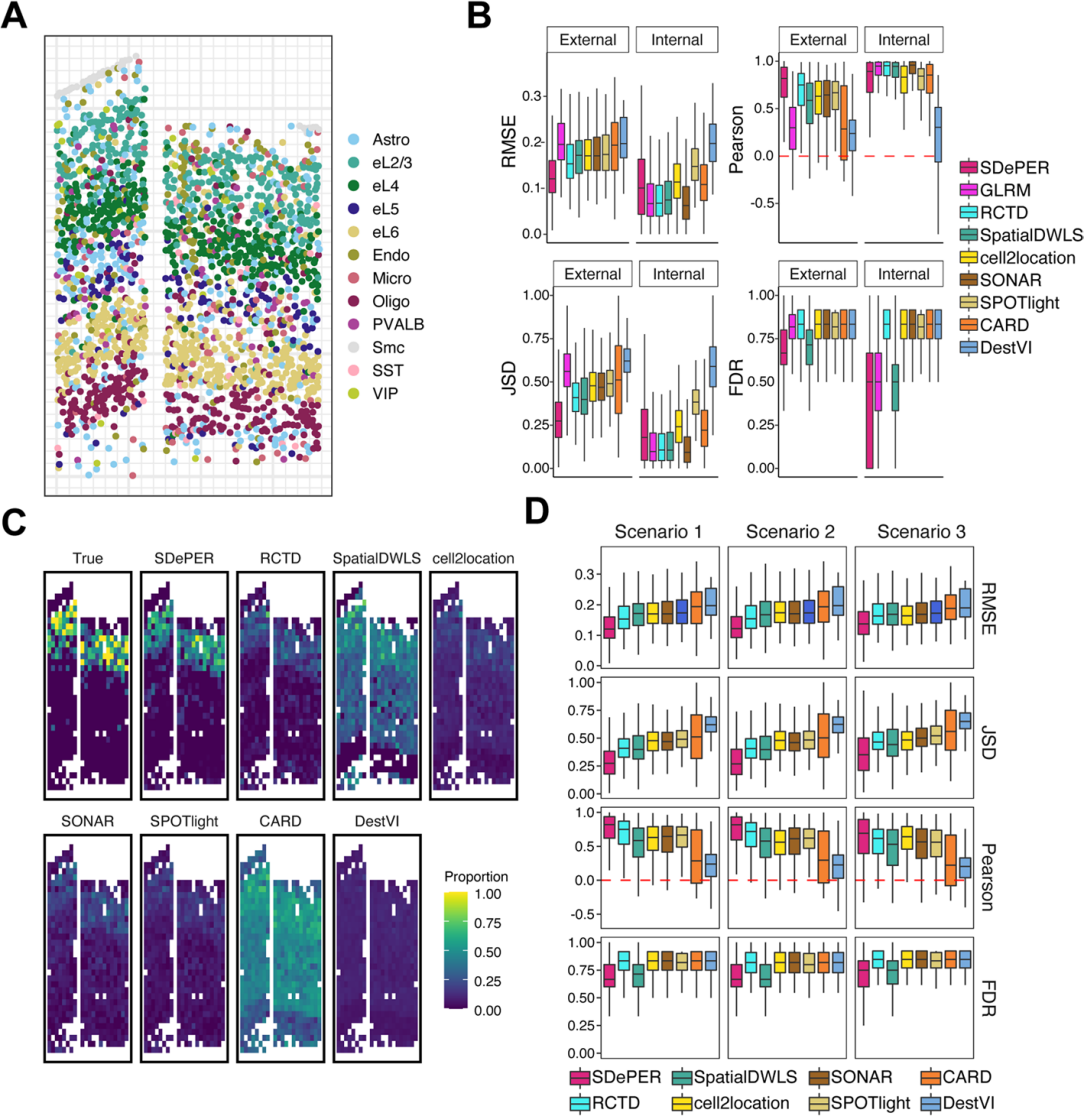
Performance evaluation and comparison using simulation studies. **A** Coarse-graining procedure to simulate ST data (581 spots) with ground truth. **B** Demonstration of the impact of platform effects on method performance: boxplots show the median (center line), interquartile range (hinges), and 1.5 times the interquartile (whiskers) of RMSE, JSD, Pearson’s correlation, and FDR across all 581 spots using external scRNA-seq reference and internal single-cell level spatial reference. **C** The proportion of L2/3 excitatory neurons in the simulated spots. **D** Boxplots show the median (center line), interquartile range (hinges), and 1.5 times the interquartile (whiskers) of RMSE, JSD, Pearson’s correlation, and FDR across 581 spots using different scRNA-seq reference: scenario 1: scRNA-seq reference with matched cell type; scenario 2: one missing cell type in scRNA-seq reference; scenario 3: one added irrelevant cell type in scRNA-seq reference

We conducted simulations to evaluate the performance of SDePER and compared it to seven other deconvolution methods with the best performance based on previous benchmarking studies [11, 29–31]: RCTD [12], SpatialDWLS [26], cell2location [15], SONAR [28], SPOTlight [25], CARD [24], and DestVI [14]. ST data with 581 spots was simulated by coarse-graining a real spatial transcriptomic data with single-cell resolution (Fig. 2A) generated using the STARmap technology [35]. The true cell-type composition at each simulated spot is calculated and serves as the ground truth. To demonstrate the impact of platform effects [12] on the method performance, each method was applied using both external and internal reference data, representing situations with and without platform effects. Moreover, to demonstrate the effectiveness of CVAE on removing platform effects, we ran SDePER with the CVAE component deactivated, which was named GLRM.

Performance comparison based on the median RMSE, Pearson correlation, and JSD showed that SDePER achieved the highest estimation accuracy regardless of the existence of platform effects (Fig. 2B, Additional File 1: Fig. S1). Visualization of the ground truth and estimated proportion of L2/L3 excitatory neurons (Fig. 2C) and other cell types (Additional File 1: Fig. S2) using an external reference further confirmed the highest accuracy of SDePER results (Pearson correlation = 0.872). Furthermore, the accuracy of all methods was lower for external reference compared to internal reference, indicating that platform effects have a complicated form that cannot be efficiently addressed using a random effect. SDePER and DestVI had the smallest accuracy difference between internal and external reference, suggesting their best robustness to platform effects (Fig. 2B, Additional File 1: Fig. S1). Lastly, when using internal reference without platform effects, SDePER had slightly worse performance than GLRM with an increase of 0.034 and 0.082 in the median RMSE and JSD, respectively, and a decrease of 0.056 in the median correlation, indicating the potential noise introduced by the CVAE component. But when platform effects were present (external reference), SDePER had a much better performance than GLRM (39%, 51%, 174%, 19% improvement in RMSE, JSD, Pearson’s correlation, and FDR, respectively), and this increase was much larger than the decrease in performance when using internal reference (Additional File 1: Table S1). All these demonstrated that SDePER achieved the best performance in both estimating cell-type compositions and removing platform effects.

To demonstrate the performance of SDePER in datasets with both reference and ST data being purely sequencing-based for small platform effects, we generated another sequencing-based simulated ST data. We retained the spatial location of cells in the STARmap data but replaced the expression profiles of each cell with those of a randomly chosen cell of the same type from an independent scRNA-seq data. All methods achieved better performance than the STARmap-based simulated data as expected, and SDePER remained to have the best performance (Additional File 1: Fig. S3).

We noticed that certain tissues, like the solid cancer tissues, may have higher cell density than the STARmap data. Therefore, we conducted simulations for high cell density. Based on all performance criteria, SDePER had robust performance across all cell density settings while GLRM had decreasing performance when the cell density increased (Additional File 1: Fig. S4) even when there were no platform effects (internal reference). This suggests that ST data with higher cell density have larger systematic difference from the reference data, no matter whether there are platform effects or not, potentially because ST data with higher cell density tend to have more variation caused by the heterogeneity across cells from the same type. CVAE successfully addressed for this difference and made SDePER robust to cell density.

### Ablation test

To understand the contribution of different components in SDePER, we conducted ablation tests by disabling each component. The results for external reference when using the STARmap-based simulated dataset (Additional File 1: Fig. S5) showed that CVAE had the most contribution and pseudo-spot inclusion in the CVAE training had the second largest contribution to the performance. Both the adaptive LASSO penalty and graph Laplacian penalty had negligible contribution to the RMSE but did contribute to lower the false discovery rate with the adaptive LASSO penalty having a slightly larger contribution. This reduction in FDR by the adaptive LASSO was also observed in simulation with only five cell types included in both ST and scRNA-seq data (Additional File 1: Fig. S6) indicating the necessity to include the LASSO penalty even when there are small number of cell types. For internal reference, i.e., when there are no platform effects, all components did not have noticeable contribution except that CVAE introduced noises and led to larger RMSE. When the sequencing-based simulated dataset was used (Additional File 1: Fig. S7), similar contribution was observed for each component but at a much smaller scale when the number of cells per spot is the same (1 ×) because the sequencing-based simulated data was expected to have smaller platform effects than the STARmap-based simulated data. But when the number of cells per spot increased, the contribution of CVAE and pseudo-spot inclusion significantly increased. This observation was consistent between the external and internal references.

Taken together, the ablation test showed that the most contributing component in SDePER is the CVAE component, which removes platform effects. It also showed that although this component did not help when there were no platform effects, it would have a large contribution when cell density is high.

### Robustness of methods to mismatching cell types

To demonstrate the robustness of methods to mismatching cell types between the reference and ST data, we conducted deconvolution under three scenarios representing perfect match, one missing cell type, and one extra cell type in the external reference data compared to the ST data. The performance rankings of all methods were consistent across these three scenarios with SDePER consistently achieving the best accuracy (Fig. 2D). In scenario 1, the improvements in RMSE of SDePER compared to RCTD, SpatialDWLS, cell2location, SONAR, SPOTlight, CARD, and DestVI were 22%, 30%, 30%, 30%, 31%, 38%, and 39%, respectively. In scenarios 2 and 3, compared to the other methods, SDePER achieved 21–38% and 15–27% improvement in RMSE, respectively. Compared to scenario 1, SDePER also had an increase of 0.002 and 0.018 in the median RMSE in scenarios 2 and 3, respectively. These results showed that SDePER had the best robustness to the mismatching cell types between the ST data and reference scRNA-seq data.

### Robustness of SDePER to rare cell types

Rare cell types in both reference scRNA-seq and ST data pose challenges to the task of deconvolution. To assess the robustness of SDePER to rare cell types, we conducted two simulation analyses for rare cell types. In the first analysis to simulate rare cell types in the reference data, the performance of SDePER on Oligodendrocytes was evaluated (Additional File 1: Fig. S8) using RMSE, false negative rate (FNR), and false discovery rate (FDR). For external reference, the performance is robust to the number of Oligodendrocytes in the down-sampled reference data. For internal reference, when there were more Oligodendrocytes, the median RMSE remained unchanged with shorter interquartile range suggesting a more stable result when there were more Oligodendrocytes. In the second simulation analysis for rare cell types in the ST data, within each group of spots with the same total number of cells, both the relative absolute error (RAE) and the false negative rate (FNR) decreased when the number of oligodendrocytes per spot increased (Additional File 1: Fig. S9). Specifically, when there were at least three cells in the spot, the rare cell type could be always identified as present (FNR = 0). When there were only two cells in the spot, SDePER had over 87% chance to identify the rare cell type as present (FNR = 0.125). This trend is consistent across spot groups with different total number of cells and between external and internal references. In summary, these results suggested that the performance of SDePER is robust to rare cell types in the reference scRNA-seq data but worse for the rare cell types in the ST data, especially when there are less than two cells in the spot.

### Mouse olfactory bulb data

**Fig. 3:**
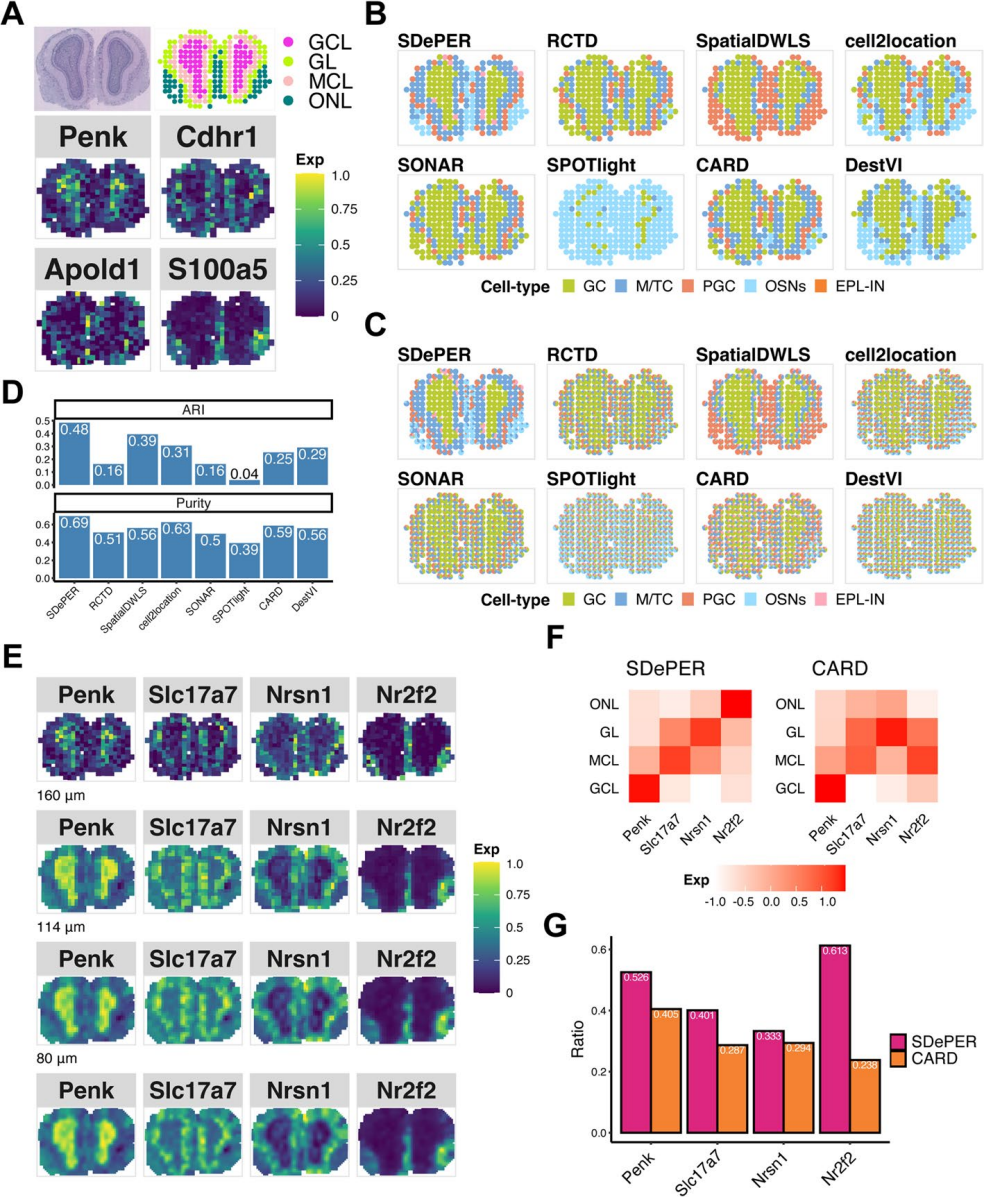
Performance evaluation and comparison using MOB dataset. **A** H&E staining of MOB (top-left), annotated regions (top-right GCL: granule cell layer; MCL: mitral cell layer; GL: glomerular layer; ONL: olfactory nerve layer) and expression pattern of cell-type-specific marker genes for dominant cell types (bottom, *Penk* for GC, *Cdhr1* for mitral and tufted cell (M/TC), *Apold1* for periglomerular cell (PGC), and *S100a5* for olfactory sensory neurons (OSNs)). **B** Visualization of inferred dominant cell type in each spot (EPL-IN: external plexiform layer interneuron). **C** Spatial scatter pie chart of estimated cell-type composition within each spot. **D** Comparing deconvolution methods using ARI (left) and purity (right). **E** Expression patterns of the corresponding layer-specific marker genes and imputed expression at three different resolution levels: 160 μm (about 64% of original size), 114 μm (about 32% of original size), 80 μm (about 16% of original size). **F** Heatmap showing average imputed expression of region-specific marker genes at 80 μm level within each annotated region for SDePER and CARD. **G** Bar plot showing the ratio of average layer-specific marker gene expression in the corresponding layer among all layers

To demonstrate the efficacy of SDePER on real data, we first applied SDePER and the other seven methods to a ST data of mouse olfactory bulb (MOB) [4] with well-defined anatomic layers organized in a well-characterized spatial architecture. We took an independent scRNA-seq data of the same tissue type profiled using the 10 × Genomics Chromium platform as reference data for the deconvolution [36] (Additional File 1: Fig. S10). Based on the H&E staining, four major tissue layers were identified from inside to outside with each dominantly composed of one cell type: the granule cell layer (GCL), mitral cell layer (MCL), glomerular layer (GL), and olfactory nerve layer (ONL) dominated by GC, M/TC, PGC, and OSNs, respectively (Fig. 3A) [4, 24]. Expression maps of marker genes for these four dominant cell types were consistent with the four annotated layers (Fig. 3A). Expression maps of marker genes for other cell types can be found in Additional File 1: Fig. S11.

The H&E staining image-based annotation and expression maps of the four dominant cell-type marker genes were considered as ground truth. The predicted dominant cell type by SDePER showed remarkable similarity with the ground truth (Fig. 3A, B). RCTD and SONAR mislabeled ONL as GCL. SpatialDWLS and DestVI did not separate ONL and GL. CARD and cell2location showed blurry layer boundaries and did not find ONL accurately. SPOTlight failed to identify the annotated regions and identified almost all spots to be dominantly OSN, potentially due to the randomness and bias introduced by its cell down-sampling procedure. Quantitative assessment of the similarity between the predicted dominant cell type and H&E staining image-based annotated layers using ARI and purity (Fig. 3D) confirmed the best performance of SDePER. In addition, when comparing the predicted dominant cell type (Fig. 3B) to the predicted cell compositions in the pie chart (Fig. 3C) for each method, SDePER showed the highest similarity between the two plots indicating less non-specific cell-type detection potentially due to its sparsity regularization.

To demonstrate the imputation results, we selected four layer-specific marker genes, one gene for each layer, from the ST data. Visualization of the original and imputed layer-specific marker gene expression on the original spatial map and three spatial maps with higher resolution (Fig. 3E) showed an expression enrichment of each layer marker gene in its corresponding layer. We compared the imputed cell-type proportion and layer-specific marker gene expression on various resolutions with CARD (Additional File 1: Fig. S12-13). To quantitatively assess the expression enrichment, we calculated the average imputed expression levels of each layer marker gene in its corresponding layer at 80 µm resolution. The average imputed expression by SDePER (Fig. 3F) displayed higher diagonal values and lower off-diagonal values, indicating a better separation between different layers based on the imputed expression than CARD. We further calculated the ratio of the average expression of each layer-marker gene in its corresponding layer to that across all layers for a quantitative assessment of the imputed expression-based layer separation (Fig. 3G). SDePER achieved a higher ratio for all four layer-marker genes than CARD, demonstrating a higher accuracy in imputed expression.

### Stage III cutaneous malignant melanoma data

**Fig. 4:**
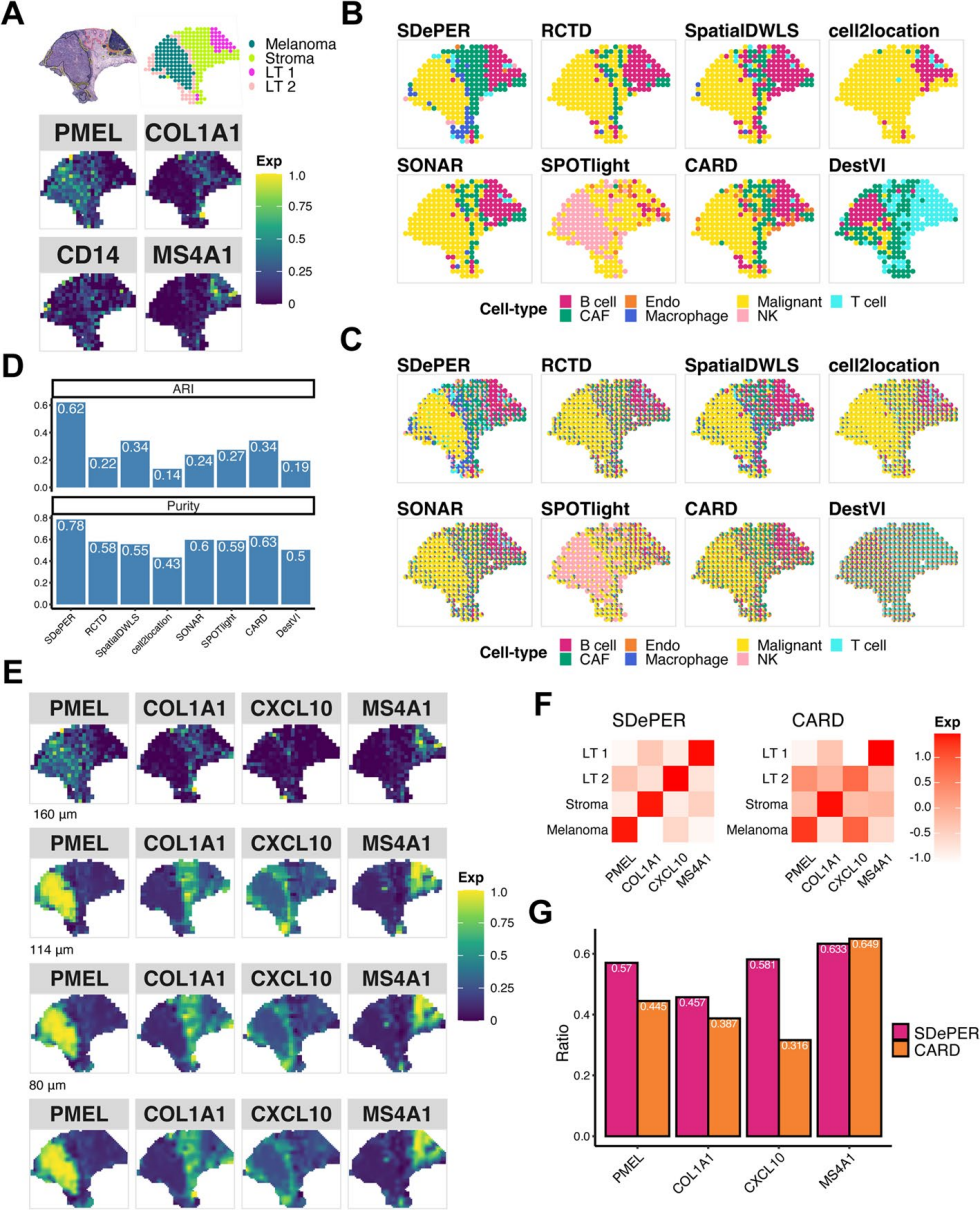
Performance evaluation and comparison using melanoma dataset. **A** H&E staining of melanoma (top left, melanoma (black), stroma (red), lymphoid tissue (yellow)), annotated regions (top right, LT lymphoid tissue) based on BayesSpace and expression pattern of cell-type-specific marker genes for dominant cell types (bottom, *PMEL* for malignant melanoma regions, *COL1A1* for fibroblast in stroma regions, *CD14* for macrophage, and *MS4A1* for B cells). **B** Visualization of inferred dominant cell type in each spot (CAF cancer-associated fibroblasts, Endo endothelial, NK natural killer). **C** Spatial scatter pie chart of estimated cell-type composition within each spot. **D** Comparing deconvolution methods using ARI and purity. **E** Expression patterns of the corresponding region-specific marker genes and its imputed expression at three different resolution levels: 160 μm (about 64% of original size), 114 μm (about 32% of original size), 80 μm (about 16% of original size). **F** Heatmap showing average imputed expression of region-specific marker genes at 80 μm level within each annotated region for SDePER and CARD. **G** Bar plot showing the ratio of average layer-specific marker gene expression in the corresponding layer among all layers

The second real data we analyzed investigated the cutaneous malignant melanoma sample from the lymph nodes [7]. Manual annotation of the tissue slide using H&E staining and clustering analysis of the ST data (Fig. 4A) identified regions of melanoma, stroma, and lymphoid tissue with expected cell types [7, 33]. For each expected cell type in each region, we selected its marker genes from existing literature [37], which included *PMEL* for malignant cells in melanoma regions, *COL1A1* for fibroblast in stroma regions, *MS4A1* for B cells and *CD14* for macrophage in lymphoid tissues [37]. The expression map of these marker genes in ST data (Fig. 4A) confirmed the prevalence of fibroblasts in stroma regions, B cells in the right-top lymphoid tissue 1, and macrophages in the lymphoid tissue 2 surrounding the melanoma region. Expression maps of marker genes for other cell types can be found in Additional File 1: Fig. S14. We used an independent scRNA-seq data of untreated metastatic melanoma samples from human lymph nodes [38] profiled using the inDrop technology as the reference data for deconvolution (Additional File 1: Fig. S15).

Like the results of MOB data, the dominant cell type predicted by SDePER highly matched the H&E staining image-based annotation (Fig. 4B). In contrast, other methods failed to identify a clear boundary between regions (Fig. 4B). SDePER also achieved the highest ARI and purity that are 1.82 and 1.24 times, respectively, as high as the second-best method (Fig. 4D). The least non-specific cell-type detection by SDePER was again observed (Fig. 4B, C).

Four region-specific marker genes (*PMEL* for melanoma region, *COL1A1* for stroma region, *CXCL10* for lymphoid tissue 2, and *MS4A1* for lymphoid tissue 1) were identified from the ST data (Fig. 4E) to demonstrate the accuracy of imputed expression. As expected, the imputed expression map of each region marker gene by SDePER showed increased expression in the correct region (Fig. 4E). Compared to CARD, the SDePER recovered the cell-type proportion at a higher resolution (Additional File 1: Fig. S16) and imputed *CXCL10* expression remarkedly better resembled the lymphoid tissue 2 on the periphery of the tumor (Additional File 1: Fig. S17). The average imputed expression of each region marker gene by SDePER had a better enrichment for the correct region (Fig. 4F), confirmed by the higher expression ratio of SDePER for each region marker gene (Fig. 4G). All these results suggested that the SDePER-imputed gene expression was more accurate.

### HER2-positive breast tumor data

**Fig. 5:**
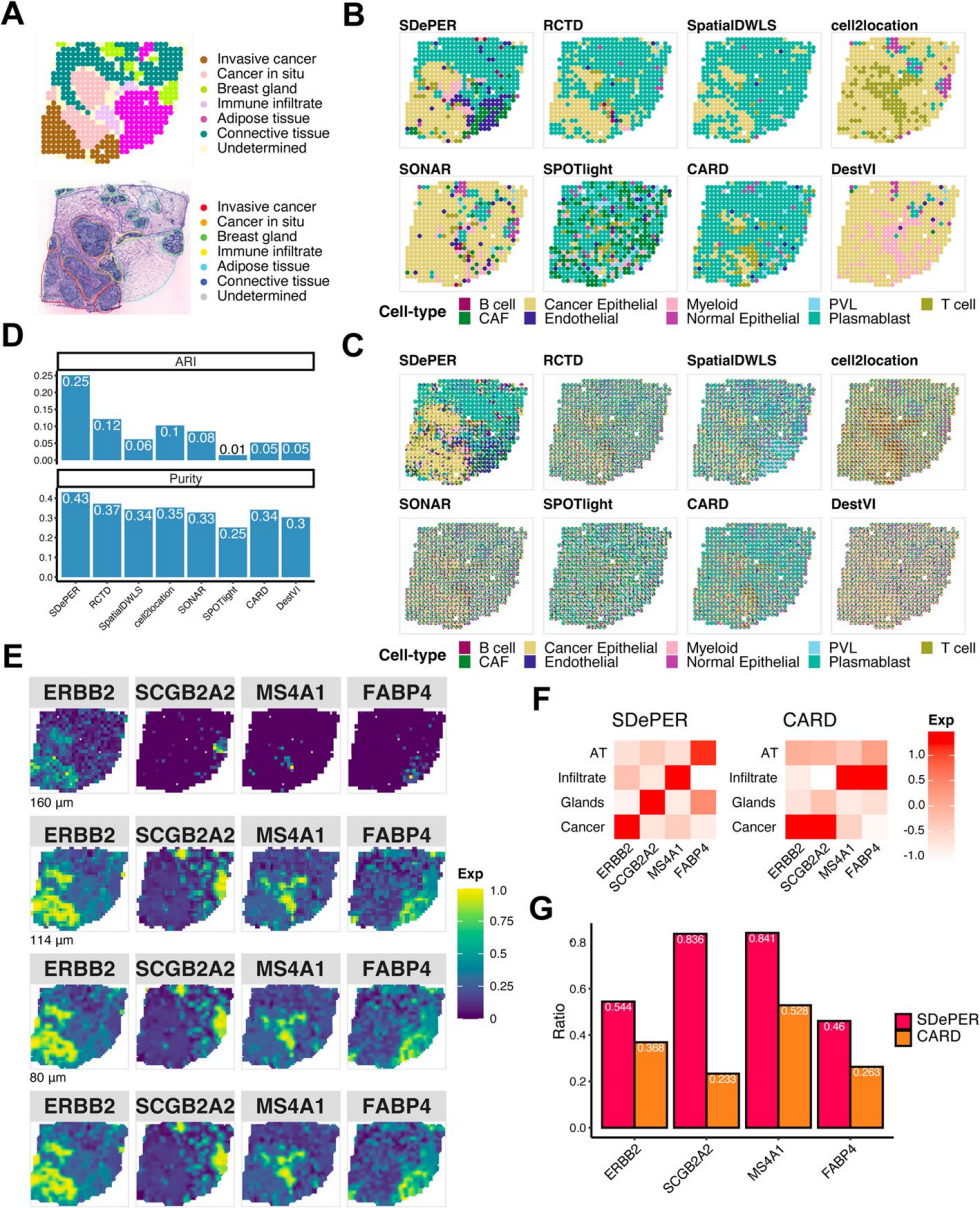
Performance evaluation and comparison using breast cancer dataset. **A** H&E staining of breast cancer and annotated regions. **B** Visualization of inferred dominant cell type in each spot (CAF cancer-associated fibroblasts, PVL perivascular-like). **C** Spatial scatter pie chart of estimated cell-type composition within each spot. **D** Comparing deconvolution methods using ARI (left) and purity (right). **E** Expression patterns of the corresponding region-specific marker genes and its imputed expression at three different resolution levels: 160 μm (about 64% of original size), 114 μm (about 32% of original size), 80 μm (about 16% of original size). **F** Heatmap showing average imputed expression of region-specific marker genes at locations in each annotated region for SDePER and CARD (AT, adipose tissue; Infiltrate, immune infiltrate; Glands, breast glands; Cancer, invasive cancer and cancer in situ). Imputation at 80 μm level was used. A red diagonal indicates that each region-specific marker gene was imputed to have high expression in the region that it is the marker for and low expression in the other regions for which it is not a marker for. **G** Bar plot showing the ratio of the average imputed expression levels of the region-specific marker gene in the region that it is a maker for to the other regions. Higher ratio corresponds to more different imputed expression levels of the marker genes between its represented region and other regions

Next, we analyzed the ST data from patients with HER2-positive breast tumor, which consists of various cell types arranged in spatial domains annotated by pathologists [8]. The annotated regions included two cancer regions (cancer in situ and invasive cancer), four named regions (adipose tissue, breast glands, connective tissue, and immune infiltrate), and undetermined regions (Fig. 5A). Although no expected cell type was provided in each region, we expect the cancer regions to enrich for cancer epithelial cells. The expression map of identified cell markers for each cell type confirmed our hypothesis (Additional File 1: Fig. S18). An external scRNA-seq dataset from 5 HER2-positive patients was used as the reference data [39] (Additional File 1: Fig. S19).

The SDePER predicted dominant cell type had the best resemblance to the boundaries between tumor and normal regions in the H&E staining image (Fig. 5B), while other methods failed to detect regions annotated in the staining image. SPOTlight failed to detect any cancer epithelial in the cancer regions, whereas SONAR, DestVI, and cell2location predicted almost all spots to be mainly cancer epithelial cells, which was inconsistent with the H&E staining image. RCTD, SpatialDWLS, and CARD had vague boundaries between tumor and normal regions. SDePER also achieved the highest ARI and purity, which were 2.08 and 1.16 times as high as the second-best method, respectively, confirming the best performance of SDePER (Fig. 5D). The least non-specific cell types were detected by SDePER (Fig. 5B, C).

For the imputation results, we identified four region-specific markers from the ST data for the four annotated regions: cancer region, breast glands, immune infiltrate, and adipose tissue. The imputed expression map of each gene by SDePER showed a more refined and accurate boundary for its corresponding region (Fig. 5E). Compared to CARD, the SDePER imputed cell-type proportion and expression of each region marker gene separated its corresponding region from the other regions better demonstrated by visualization (Additional File 1: Fig. S20-21). Quantitative measure also confirmed that SDePER had a higher enrichment with a larger expression ratio (Fig. 5F–G). Furthermore, imputed expression of *ERBB2* showed an increase in the cancer region matching previous literature [40]. The expression map of known plasma cell marker gene (Additional File 1: Fig. S18), *IGKC*, also ascertained the prevalence of plasma cells in breast glands and connective tissue as predicted by SDePER, rather than perivascular-like (PVL) cells predicted by RCTD. This was also confirmed by the original paper [8]. These further confirmed the higher accuracy of imputation by SDePER.

In the original publication for the breast cancer ST data [8], the co-localization of B cells and T cells was shown to be predictive of the tertiary lymphoid-like structure (TLS) presence in the tissue slice. Visualization of the cell-type proportion estimated by SDePER (Additional File 1: Fig. S22) also demonstrated the co-localization in the TLS regions. In addition, SDePER results also showed enrichment of myeloid cells in the TLS regions which is supported by previous literature [41–43].

### Idiopathic pulmonary fibrosis lung data

**Fig. 6:**
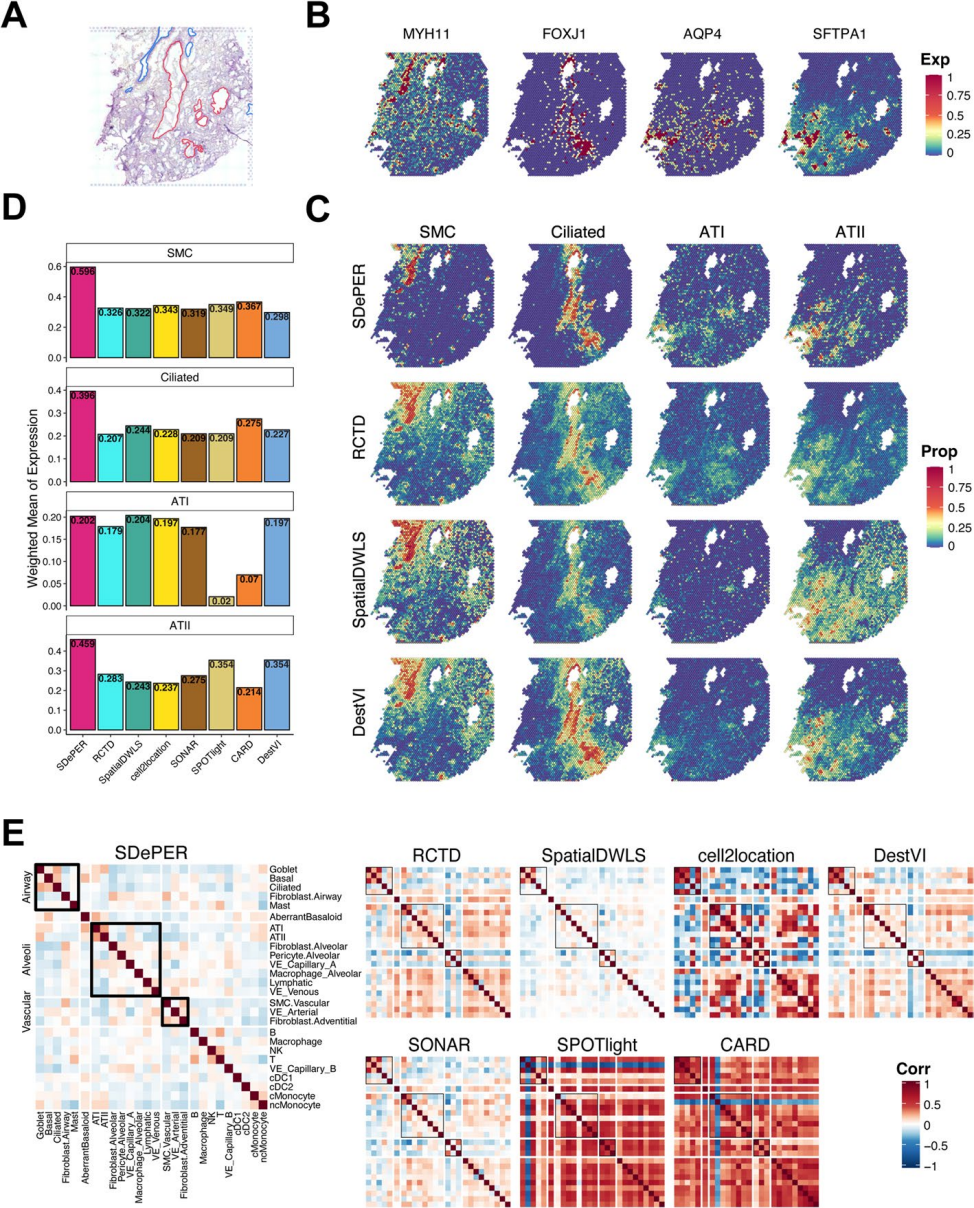
Performance evaluation and comparison using idiopathic pulmonary fibrosis lung dataset. **A** H&E staining of breast cancer with annotated regions: respiratory airway (red) and blood vessels (blue). **B** Heatmaps of selected cell-type marker genes expression patterns for SMC (MYH11), ciliated cells (FOXJ1), AT1 (AQP4), and AT2 (SFTPA1) cells. **C** The estimated cell-type proportions on each location for SMC, ciliated cells, AT1, and AT2 cells inferred by SDePER, RCTD, SpatialDWLS, and DestVI. **D** Barplot of the average expression of marker genes among all spots weighted by estimated proportions of the corresponding cell type for each method. **E** Pairwise correlation of estimated cell-type proportions for each method

Lastly, we generated the ST data of a frozen human explant lung sample with idiopathic pulmonary fibrosis (IPF), using the 10 × Genomics Visium platform. IPF is a progressive and irreversible, scarring, and fibrotic lung disease that leads to a complete remodeling of the lung architecture. Due to the complexity and distortion of lung architecture in fibrotic frozen tissues, only the respiratory airway and blood vessels were confidently annotated by a lung fibrosis expert pathologist (Fig. 6A). For deconvolution, we utilized the scRNA-seq dataset of IPF distal lung parenchyma sample as the reference data [44] (Additional File 1: Fig. S23).

We demonstrated the results using four cell types: ciliated cells from the airway, smooth muscle cells (SMC) from the vascular and alveolar type 1 (AT1) and type 2 (AT2) cells from the alveoli. Expression maps of marker genes for ciliated cells and SMC match the annotations of airway and vascular, respectively (Fig. 6B). Marker gene expression maps of AT1 and AT2 cells also suggested their prevalence over the distal side of the lung where alveoli are located.

Visualization of the predicted cell-type proportions (Fig. 6C, Additional File 1: S24) showed that SDePER captured the location of four cell types accurately and precisely, which well-matched both the expression map of marker genes (Fig. 6B) and the pathological annotation (Fig. 6A). But other methods either lacked the specificity in the estimation or failed to identify cell types. RCTD, SpatialDWLS, and DestVI had excessive non-zero estimations in spots lacking the corresponding marker gene expression, especially for SMC in the vascular region and ciliated cells in the airway. For each method, the average expression of each marker gene across all spots weighted by the predicted proportion of its corresponding cell type was calculated to quantitatively measure the consistency between the estimated cell-type compositions and marker gene expression maps. SDePER achieved the highest weighted mean for SMC, ciliated cells, and AT2 cells (Fig. 6D). It had a comparable performance in AT1 cells. These quantitatively confirmed the highest estimation accuracy of SDePER. Moreover, SDePER results demonstrated co-localization of AT1 and AT2 cells on the margin of tissue slide with the highest pairwise correlation of estimated cell-type proportions, which is consistent with the cell-type marker gene expression map and anatomy of human lungs (Fig. 6E).

Furthermore, we examined the results of other important cell types (Additional File 1: Fig. S25), including aberrant basaloid cells, adventitial fibroblast, and airway fibroblast. Aberrant basaloid cells seem to co-localize well with basal cells. They are also present in the alveoli together with AT I and AT II cells. Adventitial fibroblast was found to co-localize with the vascular smooth muscle cells, suggesting its presence in the vascular compartment. A recent spatial transcriptomic study [45] of IPF lung using the 10 × Genomics Xenium platform validated this finding. The airway fibroblasts were found to be present in the vascular compartment instead of in the airway, indicating that a further investigation of the location of these cells in human lungs is needed.

Overall, this is the first time that ST data from the human lung sample is used for the demonstration of cell-type deconvolution. The results showed that SDePER is a reliable method for complex tissue samples with vague structures and rare cell types.

## Discussion

There are several directions of extensions of SDePER. First, SDePER removes the systematic differences between ST and scRNA-seq data in an unsupervised manner, which can be improved by utilizing the known cell-type compositions of reference scRNA-seq data and pseudo-spot data as supervision in the training of CVAE. Multi-task learning strategy can be used to integrate the unsupervised and supervised learning and leverage the information of cell-type compositions in the pseudo-spot data to guide the CVAE training. Second, we assumed that the distribution of embeddings in the CVAE latent space follows a standard normal distribution. This assumption can be relieved by introducing importance sampling [46, 47]. Third, the encoder and decoder in CVAE are multi-layer neural networks, which are generic to approximate any functions [48, 49], leading to its relatively high variance and sensitivity to the variation of model structure and initialization. This can be improved by using a negative binomial distribution [50] instead in the decoder. Fourth, the computational speed of SDePER may be a concern for larger-scale ST data with tens of thousands of spots. Based on the SDePER model and algorithm, the computational time should linearly increase with the total number of spots. We further evaluated how the number of genes used in the CVAE training and GLRM model fitting affected the computational speed (Additional File 1: Fig. S26), which demonstrated a linearly increasing computational time when the number of genes increased. For large-scale spatial transcriptomics data, by selecting limited but representative genes (~ 500 genes), we could finish the analysis of one 10 × Visium tissue slide with about around 3500 spots in ~ 2.5 h. For data with even larger scale, it is possible to disable the graph Laplacian penalty in SDePER so that it can be run parallelly across different spots. In addition, the computational efficiency of SDePER can be further improved by caching the calculated log-likelihoods in GLRM fitting to avoid repetitive. Finally, results for the internal reference showed that CVAE may introduce noise when there were no platform effects. One potential way to assess the severity of platform effects is to examine the overlap between reference and real ST data in the UMAP before and after the CVAE process. This is similar to the integration analysis which improves overlap between cells of the same type across batches. Larger improvement in the overlap between the two platforms may suggest more severe platform effects.

## Conclusions

We have developed a novel deconvolution method, SDePER, to deconvolute spatial barcoding-based transcriptomic data using reference scRNA-seq data, with considerations of platform effects, sparsity, and spatial correlation. Through simulations, we demonstrated the superior performance and robustness of SDePER to platform effects and mismatching cell types between ST and reference data. Applications to datasets from various tissue types, species, and platforms also showed a superior accuracy in the estimated cell-type compositions and imputed gene expression of SDePER.

## Methods

### SDePER method overview

SDePER is built upon the combination of a conditional variational autoencoder (CVAE) [34] and a graph Laplacian regularized regression model (GLRM). The CVAE component aims to remove platform effects and the GLRM component aims to estimate cell-type compositions at each spot based on cell-type-specific signatures from reference scRNA-seq data with considerations of sparsity and spatial correlation of cell-type compositions between neighboring spots in the tissue. Based on the estimated cell-type proportions, the imputation of cell-type compositions and gene expression at unmeasured locations in refined spatial maps with higher resolution is performed using a nearest neighbor random walk.

### Conditional variational autoencoder for platform effect adjustment

The CVAE model [34] considers the two technology platforms, i.e., reference scRNA-seq and ST, as two conditions. The loss function of CVAE is defined as

$$\text{loss}=-KL({q}_{\phi }({\varvec{z}}|{\varvec{x}},c)\boldsymbol{ }|\left|{p}_{\omega }\left({\varvec{z}}\right)\right)+ {\mathbb{E}}_{{\varvec{z}}}\left(\text{log}\left({p}_{\omega }\left({\varvec{x}}|{\varvec{z}},c\right)\right)\right),$$

where ${\varvec{x}}$ represents the gene expression profile, $c$ is the conditional variable, ${\varvec{z}}$ is the latent embedding in the latent space, ${q}_{\phi }$ is the encoder parameterized by $\phi$ to embed samples into the latent space, ${p}_{\omega }({\varvec{z}})$ is the prior distribution of latent embedding ${\varvec{z}}$ defined as the standard Gaussian distribution $\mathcal{N}(0, {\varvec{I}})$, ${p}_{\omega }({\varvec{x}}|{\varvec{z}},c)$ is the decoder parameterized by $\omega$ to generate gene expression data given the latent embedding ${\varvec{z}}$ and conditional variable $c$, and KL is the Kullback-Leibler divergence function. The CVAE loss function is optimized using Adam [51], which will learn ${q}_{\phi }({\varvec{z}}|{\varvec{x}},c)$, ${p}_{\omega }({\varvec{x}}|{\varvec{z}},c)$ and ${\varvec{z}}$ from the data.

Since CVAE assumes a Gaussian distribution for data under both conditions, it is critical for the data under the scRNA-seq condition to cover a similar spectrum of cell-type proportions as the ST data. However, the reference scRNA-seq data only has data with one single-cell type, whereas each spot in the ST data can have cells from multiple cell types. To make the training data under the two conditions have a similar spectrum of cell-type compositions, pseudo-spot data are generated from the reference scRNA-seq data to provide a wide spectrum of cell-type compositions for the input under the scRNA-seq condition. For each pseudo-spot, we randomly select a set of cells from reference scRNA-seq data and calculate the average normalized gene expression across cells as the expression profile for the pseudo-spot. The range of the number of selected cells per pseudo-spot is specified based on the cell density in real ST data. In total, the number of pseudo-spots generated is $\text{min}(100\times N\times K, \text{500,000})$, where $N$ is the number of spots in the real ST data and $K$ is the number of cell types in the reference scRNA-seq data. We train the CVAE model using 80% of the pseudo spots, the reference scRNA-seq data, and real ST data. The rest 20% pseudo-spots are used as validation data for learning rate decay and early stopping. Genes used in CVAE are the union of top highly variable genes and cell-type marker genes identified from the reference scRNA-seq data identified using Scanpy 1.9.1 [52]. The sizes of both gene lists can be tuned by users based on the properties of reference scRNA-seq data. Because the training of CVAE is sensitive to differences in data range across different genes, the normalized expression of each gene is further rescaled separately for the scRNA-seq and ST condition to be from 0 to 10 using min–max scaling. The conditional variable in CVAE represents which platform (scRNA-seq or ST) generated the data and is set to 0 or 10 by default. In the training data, the conditional variable for real ST data was set to 10 for the ST condition and the conditional variable for the pseudo-spot data and reference scRNA-seq data was set to 0 for the scRNA-seq condition.

In the CVAE training, we set the number of neurons in latent space as three times the number of cell types in the reference scRNA-seq data and use one hidden layer for both encoder and decoder under each condition, in which the number of neurons is the largest integer no more than the geometric mean of the number of neurons in the input layer and latent space. We use Adam [51] for optimization and the initial learning rate is set to 0.003 with decay specified based on the value of loss function of the validation dataset. The number of epochs is set to 1000 and the early training stopping criteria is when the value of loss function of the validation dataset increases.

After the CVAE training, the real ST data is embedded into the latent space for the ST condition (conditional variable = 10) and then is decoded using the decoder for the scRNA-seq condition (conditional variable = 0). The reference scRNA-seq data is encoded and decoded for denoising using the encoder and decoder for the scRNA-seq condition (conditional variable = 0). The decoded gene expression levels are min–max scaled back using the scRNA-seq rescaling factors. For the real ST data, the rescaled values are further multiplied by 10,000 and rounded to the nearest integers. By using the same decoder, ST and scRNA-seq data are transformed into the same space to remove platform effects. Visualization of the transformed data showed that enough biological signals were retained to separate different cell types (Additional File 1: Fig. S27). The transformed real ST data and reference scRNA-seq data serve as input to the GLRM component. In addition, the mean–variance relationship was well preserved after the CVAE adjustment in all real datasets (Additional File 1: Fig. S28).

Multiple batch effect removal methods have been developed for scRNA-seq data including MNN [53], Harmony [54], Seurat Integration [55], and so on, which can be used to correct for platform effects as well. However, these methods strongly rely on the assumption that there are common cell types or shared biological cell states between batches. Specifically, the MNN-based approaches, such as the Seurat Data Integration method, identify pairs of cells from different batches and the difference between cells in each pair is utilized to estimate batch effects. When scRNA-seq and ST data are considered as two batches by these approaches, each spot from the ST data is a mixture of multiple cells with potentially multiple cell types while each cell from the scRNA-seq data has only one cell type. Unless there are many spots in the ST data that have only one cell type, the identified “cell pairs” do not have the same biological state and the difference between them include both platform effects and cell-type composition difference. This causes the estimated batch effects to be larger than platform effects, so these approaches cannot provide adequate adjustment for platform effects. CVAE is a deep generative model which learns the data distribution in a latent space and a generative process to generate new data points from the learned distribution. It assumes that scRNA-seq data and ST data share the same type of distribution in the embedding space, which is a weaker assumption than the batch correction methods. We accommodate for this assumption by adding pseudo-spots in the CVAE training data. The generated new data will retain the original data distribution as well as the original biological meaning so the CVAE adjusted data is still gene expression data, which is critically important for the GLRM component because it assumes a linear additive relationship between the ST and reference data. To demonstrate the advantage of CVAE, we replaced the CVAE component in SDePER with Seurat Integration method and ran it on the STARmap-based simulated data with external reference. We compared the results to those of SDePER and GLRM (Additional File 1: Fig. S29). The comparison showed that Seurat Integration did correct for a certain part of the platform effects so it had a certain improvement over GLRM. But SDePER was able to achieve further and larger improvement over Seurat + GLRM, suggesting higher efficiency of CVAE in correcting for platform effects than Seurat.

### Graph Laplacian regularized model for cell-type deconvolution

We fit a graph Laplacian regularized model to estimate cell-type compositions in each spot using the transformed ST and reference scRNA-seq data. Since biological signals can be lost by the CVAE adjustment, we identified cell-type marker genes from the transformed reference scRNA-seq data by comparing each cell type to every other cell type using the Wilcoxon Rank Sum test implemented in the FindMarker function in Seurat. Genes with false discovery rate less than 0.05, fold change ≥ 1.2, pct.1 ≥ 0.3 and pct.2 ≤ 0.1 were kept and sorted based on the fold change. By default, the top 20 genes across all comparisons were merged and used to fit the GLRM model. The transformed ST data of each gene is assumed to follow a Poisson distribution with the log-transformed mean being a linear combination of its transformed across all cell types, which forms the base model. The transformed expression profile of each cell type is calculated as the average expression profiles across cells of the given cell type from the transformed reference scRNA-seq data. On top of the base model, we incorporate the spot location information using graph Laplacian regularization that encourages cell-type compositions of neighboring spots to be similar. We also enforce cell-type sparsity within each spot using adaptive LASSO regularization. Specifically, the GLRM component consists of the following three major components.

#### Base model

The transformed ST data is modeled using a Poisson-loglinear model which considers dispersion in the data (Additional File 1: Fig. S28). For each spot $i$ and gene $j$, the transformed ST count ${Y}_{ij}$ is assumed to follow a Poisson distribution:

$${Y}_{ij}|{\lambda }_{ij}\sim \text{Poisson}\left({N}_{i}{\lambda }_{ij}\right),$$

where ${N}_{i}$ is the observed total UMI count of spot $i$ and ${\lambda }_{ij}$ represents the true underlying relative expression level of gene $j$ in spot $i$. The rate parameter ${\lambda }_{ij}$ is further modeled as a combination of expression profiles of all $K$ cell types weighted by the cell-type proportions,

$$\text{log}\left({\lambda }_{ij}\right)={\alpha }_{i}+\text{log}\left({\sum }_{k=1}^{K}{\theta }_{ik}{\mu }_{kj}\right)+{\epsilon }_{ij},$$

where ${\alpha }_{i}$ is a parameter representing spot-specific fixed effect, ${\theta }_{ik}$ is the proportion of cells from cell-type $k$ in spot $i$, ${\mu }_{kj}$ is the mean expression level of gene $j$ in cell-type $k$ calculated from the transformed reference scRNA-seq data, and ${\epsilon }_{ij}$ is a random error that follows a normal distribution with mean 0 and variance ${\sigma }^{2}$ as defined in RCTD [12]. The distribution of ${\epsilon }_{ij}$ is relaxed to include a heavy tail using an approximation to a Cauchy–Gaussian mixture distribution, which is robust to outliers [56],

$$p\left(\epsilon \right)=\left\{\begin{array}{c}\frac{C}{\surd 2\pi \sigma }{e}^{-\frac{{\epsilon }^{2}}{2{\sigma }^{2}}}, \left|\epsilon \right|\le 3\sigma \\ \frac{2\surd 2C}{9(\epsilon \sigma -\frac{7}{3}{\sigma }^{2})\surd \pi }{e}^{-\frac{9}{2}}, \left|\epsilon \right|>3\sigma \end{array},\right.$$

Where $C$ is a normalizing constant which is chosen to make $p\left(\epsilon \right)$ integrate to 1. The model parameters ${\theta }_{ik}$ are subject to the constraint that ${\sum }_{k=1}^{K}{\theta }_{ik}=1$ and ${\theta }_{ik}\ge 0$ for all $i$ and $k$. We considered ${\alpha }_{i}$ because previous studies [37, 57–60] demonstrated that the gene expression profile of cells of the same type could vary depending on where they are located in the tissue, potentially due to the influences from neighboring cells or tissue microenvironment.

#### Adaptive LASSO regularization

To enforce the local sparsity of cell types in each spot, we penalize the likelihood function of the base model using the adaptive Lasso penalty [61]. For each spot $i$, we define the adaptive Lasso penalty as

$$r\left({\theta }_{i}\right)={\sum }_{k=1}^{K}{q}_{ik}\left|{\theta }_{ik}\right|,$$

where ${q}_{ik}$ is the reciprocal of maximum likelihood estimation (MLE) of ${\theta }_{ik}$ from SDePER without the adaptive LASSO and graph Laplacian penalties (base model), serving as the weight of ${\theta }_{ik}$ in the adaptive Lasso penalty.

#### Laplacian regularization

To incorporate spatial information, we represent the physical proximity between spots using an adjacency matrix ${\varvec{A}}{=[{A}_{ij}]}_{I\times I}$. Although ${\varvec{A}}$ can be calculated using the Euclidean distance between spots, for simplicity, we use unweighted adjacency matrix throughout this article, where ${A}_{ij}$ is an indicator of whether spot $i$ and $j$ are neighbors on the tissue slide, and $I$ is the total number of spots. The graph Laplacian is defined as ${\varvec{L}}={\varvec{D}}-{\varvec{A}}$, where ${\varvec{D}}$ is a diagonal matrix with ${D}_{ii}={\sum }_{j}{A}_{ij}$, the degree of spot $i$. The graph Laplacian penalty is defined as

$$\mathcal{L}\left({\varvec{\theta}}\right)=\frac{1}{2}\sum_{s,t=1}^{K}{A}_{st}{\Vert {\theta }_{s}-{\theta }_{t}\Vert }_{2}^{2}=\text{tr}\left({{\varvec{\theta}}}^{T}{\varvec{L}}{\varvec{\theta}}\right),$$

where tr(∙) is the trace of a matrix. This penalty measures the aggregate deviation of ${\varvec{\theta}}$ between neighboring spots and therefore encourages ${\varvec{\theta}}$ to be similar across neighboring spots.

Taken together, we fit the GLRM model by minimizing the following objective function:

$$F\left({\varvec{\theta}},\boldsymbol{\alpha },{\varvec{\mu}},\boldsymbol{ }{\sigma }^{2}\right)={\sum }_{i=1}^{I}\left[{l}_{i}\left({{\varvec{\theta}}}_{i},{\alpha }_{i},{\varvec{\mu}}, {\sigma }^{2}\right)+{\lambda }_{r}r\left({{\varvec{\theta}}}_{i}\right)\right]+{\lambda }_{l}\mathcal{L}\left({\varvec{\theta}}\right).$$

The first term of the objective function is the negative log-likelihood of the base model. The second term is the local adaptive Lasso penalty that enforces cell types to be sparsely present within each spot. The third term is the graph Laplacian penalty to encourage smoothness of cell-type compositions across neighboring spots. ${\lambda }_{r}$ and ${\lambda }_{l}$ are two positive hyperparameters.

The objective function is minimized using a two-stage strategy. To provide initial values of all parameters (${\varvec{\theta}}$, $\boldsymbol{\alpha }$ and ${\sigma }^{2}$) for optimization, we calculate the gene expression profile of cell-type $k$ using the average library size normalized expression levels of all identified cell-type marker genes across all cells of type $k$, denoted as ${\widehat{\mu }}_{k}$. The maximum likelihood estimations ($\widehat{{\varvec{\theta}}}$, $\widehat{\boldsymbol{\alpha }}$, and ${\widehat{\sigma }}^{2}$) of the base model are obtained using the L-BFGS algorithm [62] in SciPy 1.8.1 [63] and serve as the initial values for optimization. In the first stage of optimization, we perform cell-type selection in each spot by minimizing the negative log-likelihood function of the base model with the adaptive LASSO penalty: ${\sum }_{i=1}^{I}\left[{l}_{i}\left({{\varvec{\theta}}}_{i},{\alpha }_{i}|\widehat{{\varvec{\mu}}}, {\widehat{\sigma }}^{2}\right)+{\lambda }_{r}r\left({{\varvec{\theta}}}_{i}\right)\right]$ using alternating direction method of multipliers (ADMM) [64]. A cutoff value of 0.001 is applied to the estimate $\widehat{{\varvec{\theta}}}$ to determine which cell types are present in each spot. In the second stage of optimization, we only include cell types selected from the first stage for each spot in the base model and minimize the negative log-likelihood function of the base model with graph Laplacian regularization using ADMM: ${\sum }_{i=1}^{I}{l}_{i}\left({{\varvec{\theta}}}_{i},{\alpha }_{i}|\widehat{{\varvec{\mu}}}, {\widehat{\sigma }}^{2}\right)+{\lambda }_{l}\mathcal{L}\left({\varvec{\theta}}\right)$. The values of the two hyperparameters, ${\lambda }_{r}$ and ${\lambda }_{l}$, are chosen using fivefold cross-validation. The cell-type marker genes identified from the transformed reference scRNA-seq data were randomly divided into five groups with equal size. Each group was considered as validation data and the rest groups were used as training datasets. For given values of ${\lambda }_{r}$ and ${\lambda }_{l}$, the training data was used to fit the GLRM model, which was used to calculate the log-likelihood of the validation data using the base model. The log-likelihood of the five validation datasets was averaged and compared across different settings of ${\lambda }_{r}$ and ${\lambda }_{l}$. The setting that achieved the largest average log likelihood was chosen.

### Imputation on cell-type composition and gene expression

SDePER borrows information from neighboring spots to perform imputation of cell-type compositions at unmeasured locations in refined tissue map with arbitrary resolution by taking the nearest neighbor random walk on the spatial graph. We first use the “finding contour” function in opencv [65] to determine the contours of the tissue shape and distinguish outlines of the tissue and holes inside the tissue. The spatial spots closest to the outline and border of holes are set to be edge spots. We also develop a custom algorithm to calculate the missing spots in the holes. Suppose the spatial coordinates of the center for spot $i$ is ${c}_{i}=({x}_{i}, {y}_{i})$ and the distance between centers of two neighboring spots is D. To construct a new spatial map at enhanced resolution, we first create the smallest rectangular region that covers all centers of original spots using $\left[{\text{min}}_{i}\left({x}_{i}\right), {\text{max}}_{i}\left({x}_{i}\right)\right]\times [{\text{min}}_{i}\left({y}_{i}\right), {\text{max}}_{i}\left({y}_{i}\right)]$. This rectangular is then gridded into squares with side length $d (d<D)$. Among all the squares, those with center located at ${c}_{{i}^{*}}=({x}_{{i}^{*}}, {y}_{{i}^{*}})$ satisfying one of the following criteria are considered as spots in the new map and imputed:

$${\text{min}}_{\left\{i=1,\dots ,N; i \text{is an inner spot}\right\}}\Vert {c}_{{i}^{*}}-{c}_{i}\Vert =\le D \text{or } {\text{min}}_{\left\{i=1,\dots ,N; i \text{is an edge spot}\right\}}\Vert {c}_{{i}^{*}}-{c}_{i}\Vert \le \frac{D-d}{2}.$$

These criteria filter out center locations that are far away from the edge and inner spot, outside the tissue slice or in biological holes. The new spatial map at enhanced resolution (square side length = $d$) consists of the spots with centers ${{\varvec{C}}}^{\boldsymbol{*}}=\left\{{c}_{{i}^{*}}\right\}$, ${i}^{*}=1,\dots , {N}^{*}$.

Let ${{\varvec{\theta}}}_{{i}^{*}}$ denote the cell-type proportions in spot ${i}^{*}$ in the new spatial map. To perform imputation, we first assign an initial value for ${{\varvec{\theta}}}_{{i}^{*}}$ by finding the nearest original spot(s) of spot ${i}^{*}$ and set ${{\varvec{\theta}}}_{{i}^{*}}^{0}$ as the average cell-type proportion among its neighbors. We construct a Gaussian kernel ${\varvec{W}}$ as follows

$${W}_{{i}^{*}{j}^{*}}=\left\{\begin{array}{c}0, {r}_{{i}^{*}{j}^{*}}>\phi \\ {e}^{-\frac{{r}_{{i}^{*}{j}^{*}}^{2}}{2{\tau }^{2}}}, {r}_{{i}^{*}{j}^{*}}\le \phi \end{array} {i}^{*},{j}^{*}=\text{1,2},\dots ,{N}^{*}\right.,$$

where ${r}_{{i}^{*}{j}^{*}}=\Vert {C}_{i*}-{C}_{j*}\Vert$ is the distance between two spots ${i}^{*}$ and ${j}^{*}$ in the new map, $\phi$ is a predefined neighborhood size within which spots contribute to the imputation of each other, and ${\tau }^{2}$ is the variance. A nearest neighbor random walk matrix is constructed as ${\varvec{M}}={{\varvec{D}}}^{-1}{\varvec{W}}$, where ${\varvec{D}}$ is a diagonal matrix with ${D}_{{i}^{*}{i}^{*}}={\sum }_{{j}^{*}}{W}_{{i}^{*}{j}^{*}}$. The imputed cell-type compositions are obtained by taking a one-step nearest neighbor random walk with the graph which can be written as

$${{\varvec{\theta}}}_{\text{imputed}}={\varvec{M}}{{\varvec{\theta}}}^{0}$$

We further impute gene expression at enhanced resolution as ${{\varvec{X}}}_{\text{imputed}}={{\varvec{\theta}}}_{\text{imputed}}{{\varvec{\theta}}}^{+}{\varvec{X}}$, where ${\varvec{X}}$ is the observed UMI counts from ST data, normalized by spot’s sequence depth, and ${{\varvec{\theta}}}^{+}$ is the Moore–Penrose inverse of ${\varvec{\theta}}$ with ${{\varvec{\theta}}}^{+}={\left({{\varvec{\theta}}}^{T}{\varvec{\theta}}\right)}^{-1}{{\varvec{\theta}}}^{T}$.

The hyperparameters in the Gaussian kernel ${\varvec{W}}$ include $\phi$ and ${\tau }^{2}.$ For a given real ST data, the hyperparameter tunning was conducted using the STARmap-based simulated data. We conducted coarse-graining procedures on the STARmap data to generate simulated ST dataset with different spot sizes varying from 100 × 100 to 1000 × 1000 with an interval of 100, which correspond to high to low resolution. In each simulated ST dataset, we know the true cell-type proportions in each spot. For a given setting of $\phi$ and ${\tau }^{2}$, we impute the cell-type proportions using the simulated dataset with spot size 1000 × 1000 to reconstruct spatial maps with different resolutions higher than 1000 × 1000 (smaller spot size). Then we compare the imputed cell-type proportions to the ground truth and calculated the average RMSE across the different higher resolution levels. The hyperparameter setting that achieved the smallest average RMSE was chosen. The search ranges for $\tau$ and $\phi$ are both 1–200 μm.

### Other deconvolution methods for comparison

Seven state-to-art spatial deconvolution methods were chosen to compare with SDePER, including RCTD (version 2.0.1) [12], SpatialDWLS (implemented in the R package Giotto, version 1.1.2) [26], SONAR (version 1.0.0) [28], SPOTlight (version 1.8.0) [25], cell2location (version 0.1.3) [15], DestVI (implemented in the python package scVI, version 1.1.3) [14], and CARD (version 1.0) [24]. We followed the tutorial on the GitHub repository of each method and used the recommended default parameter settings for the deconvolution analyses conducted in this article. When parameters are required to be set manually, we used the values suggested in the vignettes.

### Simulation studies

To evaluate the method performance and demonstrate the impact of platform effects on all deconvolution methods, we simulate spot-level ST data in multiple different ways.

#### STARmap-based simulation

We first simulated ST data based on the adult mouse primary visual cortex STARmap data that has single-cell resolution [35]. We extract experiments “20,180,410-BY3_1kgenes” and “20180505_BY3_1kgenes” and manually put them in the same spatial map with enough space in between so that cells or simulated spots from different experiments are not considered as spatial neighbors. To simulate the ST data, we gridded the tissue slide into squares with a side length of ~ 51.5 μm as capture spots, which generated 581 spots with 1 to 12 cells and an average of 3.6 cells present per spot. We only kept cells from cell types present in both STARmap data and external reference scRNA-seq data. In each spot, the proportion of cells from each cell type is calculated and serves as ground truth for performance evaluation. The simulated gene expression level of gene $j$ for a given spot that contains cells $i=1,\dots ,n$ is calculated as $nUM{I}_{j}=\lceil \frac{{\sum }_{i=1}^{n}\left(\frac{{U}_{ij}}{{\sum }_{j}{U}_{ij}}\right)}{n}\times N\rceil$, where ${U}_{ij}$ is the number of UMIs of gene $j$ in cell $i$ from the STARmap data and $N$ is a fixed scaling factor set to be 1000.

#### Sequencing-based simulation

The STARmap technology is a hybrid technology of in situ hybridization and sequencing. The protocol enriches for transcripts using hybridization techniques and the final nUMI is generated based on sequencing. To simulate ST data that are purely sequencing-based, we utilized a scRNA-seq dataset from the mouse visual cortex [66] measured using the inDrops technique (GEO accession number: GSE102827). We modified the STARmap data by retaining the spatial location of each cell but replacing its expression profile with that of a randomly chosen cell of the same type from the inDrops data. Then the same coarse-graining procedure was applied to this modified STARmap data to simulate purely sequencing-based ST data.

#### External and internal reference

To demonstrate the impact of platform effects on the method performance, each method was applied to the simulated data using two different reference scRNA-seq datasets: internal reference and external reference. The internal reference data is the original ST data with single-cell resolution so there were no platform effects. For STARmap-based simulation, the internal reference data was the STARmap data. For sequencing-based simulation, the internal reference data is the inDrops data (GEO: GSE102827). The external reference data is an independent publicly available scRNA-seq dataset. For both STARmap-based and sequencing-based simulations, the adult mouse visual cortex scRNA-seq dataset (GEO accession number: GSE115746) [67] was used as reference data. Under this case, significant platform effects were expected to exist because the simulated ST data and reference data were generated using the in situ sequencing and SMART-seq technologies, respectively. We selected 12 overlapping cell types between the external reference data and the STARmap data for deconvolution, which include astrocytes, excitatory neurons layer 2/3, excitatory neurons layer 4, excitatory neurons layer 5, excitatory neurons layer 6, endothelial, microglia, oligodendrocyte, *Pvalb*-positive cells, *Vip* inhibitory neurons, *Sst* neurons, and smooth muscle cells. In total, 2002 cells and 1020 genes were included in the STARmap data while 11,835 cells and 45,768 genes were in the external reference data.

#### Simulation for mismatching cell types

Deconvolutions using the overlapping 12 cell types between the STARmap data and external reference scRNA-seq data represent analysis scenario 1, under which cell types in the reference data match perfectly with those in the ST data. However, in practice, they can have mismatching cell types, so we modified the external reference data to demonstrate the robustness of all methods to mismatching cell types. In analysis scenario 2, we remove *Vip* inhibitory neurons (*n* = 1690) from the reference data. In analysis scenario 3, we added “high intronic” cells (*n* = 182), which are not present in the STARmap data, to the reference data.

#### Simulations for rare cell types

To assess the robustness of SDePER to rare cell types, we conducted the following two simulation analyses by choosing oligodendrocytes (“Oligo”) as the “rare cell type” for investigation. First, we down-sampled Oligo cells in the reference scRNA-seq data to a given number (5, 10, 20, and 50) for multiple times and used each down-sampled reference data to deconvolve the STARmap-based simulated ST data. In the second simulation analysis, we examined the performance of SDePER on Oligo cells using groups of spots stratified based on the number of Oligo cells per spot from the STARmap-based simulation. All the simulated spots were divided into groups based on the total number of cells (*n*) and the number of oligodendrocytes per spot. Within each group of spots with the same total number of cells, both the relative absolute error (RAE) and the false negative rate (FNR) were calculated.

#### Simulations for high cell density

To simulate ST data with high cell density, we kept the physical spot size the same as in the sequencing-based simulation but increased the total number of cells in each spot by 3 or 6 times, which corresponds to approximately 3 to 36 cells and 6 to 72 cells per spot with an average of 10.8 and 21.6 cells per spot, respectively. In each spot, the cell-type proportions remained the same but for each existing cell type, three or six times more cells were randomly selected from the inDrops without replacement to calculate the simulated spot data.

#### Simulations for small number of cell types

To evaluate the necessity of adaptive Lasso when the number of cell types is small, we removed all cells that do not belong to five chosen cell types (eL2/3, eL4, eL5, eL6, and Oligo) from the STARmap data. The same simulation procedure was conducted to simulate STARmap-based ST data. When conducting deconvolution on the simulated ST data using external reference, cells that do not belong to the five chosen cell types were also removed from the reference data.

#### Ablation tests

To understand the contribution of different components in SDePER, we conducted ablation tests by disabling the CVAE for platform effects removal, pseudo-spots inclusion in the CVAE training, adaptive LASSO penalty for sparsity, or graph Laplacian penalty for spatial correlation in SDePER. Three datasets were used including those from the STARmap-based simulation, sequencing-based simulation, and the simulation for high cell density. For each dataset, we consider the performance of SDePER as the baseline. The performance of SDePER with each component disabled was compared to the baseline performance to assess the contribution of the component. For adaptive LASSO penalty, the dataset from simulations for a small number of cell types was also used for the ablation test.

#### Performance evaluation criteria

To evaluate the method performance, we compare the cell-type compositions estimated by each method, ${\widehat{{\varvec{\theta}}}}_{i}$, to the ground truth ${{\varvec{\theta}}}_{i}$ for each spot $i$ using the root mean square error (RMSE) that quantifies the overall estimation accuracy, Jensen–Shannon Divergence (JSD) that assesses similarity between the estimated cell-type distribution and ground truth per spot, Pearson’s correlation coefficient that measures the similarity of estimation to ground truth, and false discovery rate (FDR) that measures how many cell types were falsely predicted to be present. Formulas of these criteria are as follows:

$$\text{RMSE}\left({\widehat{{\varvec{\theta}}}}_{i}\right)=\sqrt{\frac{1}{K}{\sum }_{k=1}^{K}{\left({\widehat{\theta }}_{ik}-{\theta }_{ik }\right)}^{2}}.$$

$\text{JSD}({\widehat{{\varvec{\theta}}}}_{i}\Vert {{\varvec{\theta}}}_{i})=\frac{1}{2} (KL({\widehat{{\varvec{\theta}}}}_{i}\Vert \frac{{\widehat{{\varvec{\theta}}}}_{i}+ {{\varvec{\theta}}}_{i}}{2}) + KL({{\varvec{\theta}}}_{i}\Vert \frac{{\widehat{{\varvec{\theta}}}}_{i}+ {{\varvec{\theta}}}_{i}}{2}))$, where $\text{KL}(\cdot \Vert \cdot )$ represents Kullback–Leibler divergence.

$$cor\left({\widehat{{\varvec{\theta}}}}_{i} , {{\varvec{\theta}}}_{i}\right)=\frac{{\sum }_{k=1}^{K}({\widehat{\theta }}_{ik}-{\overline{\widehat{\theta }} }_{i })({\theta }_{ik }-{\overline{\theta }}_{i})}{\sqrt{{\sum }_{k=1}^{K}{\left({\widehat{\theta }}_{ik}-{\overline{\widehat{\theta }} }_{i }\right)}^{2}}\sqrt{{\sum }_{k=1}^{K}{\left({\theta }_{ik }-{\overline{\theta }}_{i}\right)}^{2}}}.$$

$$FD{R}_{i}=\frac{{\sum }_{k=1}^{K}I({\widehat{\theta }}_{ik}\ne 0, {\theta }_{ik }=0)}{{\sum }_{k=1}^{K}I({\widehat{\theta }}_{ik}\ne 0)}.$$

### Real dataset analysis

#### Mouse olfactory bulb dataset

We obtain the mouse olfactory bulb (MOB) ST data from the Spatial Research lab [4]. We focus on the “MOB replicate 12” file which contains 16,034 genes and 282 spots. An independent scRNA-seq data is also downloaded as the reference scRNA-seq data (GEO accession number: GSE121891) [36], which consists of 18,560 genes and 12,801 cells from 5 cell types: granule cells, olfactory sensory neurons, periglomerular cells, mitral and tufted cells, and external plexiform layer interneurons.

To apply SDePER on the MOB dataset, we select 250 highly variable genes and 244 cell-type marker genes from the reference scRNA-seq data, which form a set of 434 unique genes used in the CVAE component for platform effects removal. We randomly select 10–40 single cells from the scRNA-seq data to generate a pseudo spot to mimic the number of cells per spot suggested in the MOB data; 168 cell-type marker genes are used in GLRM component. The hyperparameter ${\lambda }_{r}$ is chosen to be 1.931 and ${\lambda }_{l}$ to be 5.179 based on cross-validation. The running time of SDePER is 0.56 h in total using a 20-core, 100 GB RAM, Intel Xeon 2.6 GHz CPU machine.

#### Melanoma dataset

We download the melanoma dataset from the Spatial Research lab [7]. We focus on the second replicate from biopsy 1 because it contains regions annotated as lymphoid tissue and is extensively examined in the original paper. Biopsy 1 contains 16,148 genes and 293 spots. The reference scRNA-seq dataset is downloaded from GEO database (accession number: GSE115978), which contains 23,686 genes and 2495 cells from 8 selected samples. In total, seven cell types are present in the reference data, including malignant cells, T cells, B cells, natural killer (NK) cells, macrophages, cancer-associated fibroblasts (CAFs), and endothelial cells [38].

We choose the top 300 highly variable genes and 280 marker genes, corresponding to 534 unique genes for the CVAE component in SDePER. The number of cells per pseudo-spot is set to be 5–40 cells as provided in the original paper; 145 cell-type marker genes are used in GLRM component. The hyperparameter ${\lambda }_{r}$ is chosen to be 1.931 and ${\lambda }_{l}$ to be 37.276 based on cross-validation. The running time of SDePER is 0.56 h in total using a 32-core, 100 GB RAM, Intel Xeon 2.6 GHz CPU machine.

#### Breast cancer dataset

We obtain the HER2-positive breast cancer spatial transcriptomics dataset from a previous study [8]. The first section of patient H with 15,029 genes and 613 spots is selected for the analysis. We obtain the scRNA-seq data of five HER2-positive tumors from GEO database (accession number: GSE176078) [39] as the reference scRNA-seq data for deconvolution. The reference data consists of 29,733 genes and 19,311 cells from 9 cell types.

For the CVAE component of SDePER, we select the top 1500 highly variable genes and 824 cell-type marker genes, corresponding to 1942 unique genes. Each pseudo-spot is assumed to contain 20–70 cells based on estimation by other cancer ST studies using the same ST platform [68]; 290 cell-type marker genes are used in GLRM component. The hyperparameter ${\lambda }_{r}$ is chosen to be 1.931 and ${\lambda }_{l}$ to be 37.276 based on cross-validation. The running time of SDePER is 1.8 h in total using a 32-core, 100 GB RAM, Intel Xeon 2.6 GHz CPU machine.

#### Idiopathic pulmonary fibrotic lung dataset

We measured the ST data of a human IPF lung sample from an explanted lung of a patient with end-stage IPF explanted lung (Yale IRB:1601017047) using the 10 × s Genomics Visium platform, which is a complex and challenging sample with vague structure and different lung compartments including the bronchi, vascular, mesenchyme, and immune compartment. We selected one block of frozen lung tissue obtained from a patient with Idiopathic Pulmonary Fibrosis (IPF). Sections of 10 μm fresh frozen samples were cut from the blocks onto Visium slides (10 × Genomics) and processed according to the manufacturer’s protocol tissue sections were hematoxylin and eosin stained and finally imaged (20 ×) using a scanning microscope (EvosM700, ThermoFischer Scientific). Tissue was permeabilized and mRNAs were hybridized to the barcoded capture probes directly underneath. cDNA synthesis connects the spatial barcode and the captured mRNA. After RT and amplification by PCR, dual-indexed libraries were prepared as in the 10 × Genomics protocol and sequenced (two samples/HiSeq 6000 flow cell) with read lengths 28 bp R1, 10 bp i7 index, 10 bp i5 index, and 90 bp R2. Base calls were converted to reads with the software SpaceRanger’s implementation mkfastq (SpaceRanger v1.2.2). Multiple fastq files from the same library and strand were catenated to single files. Read2 files were subjected to two passes of contaminant trimming with cutadapt (v1.17): for the template switch oligo sequence (AAGCAGTGGTATCAACGCAGAGTACATGGG) anchored on the 5′ end and for poly(A) sequences on the 3′ end. Following trimming, read pairs were removed if the read2 was trimmed below 30 bp. Visium libraries were mapped on the human genome (10 × -provided GRCh38 reference), using STARsolo (STARsolo v2.7.6a).

This data measured the expression of 60,651 genes at 4992 spatial locations. We filtered out the spatial location not covered by tissue and the genes not expressed on all spatial locations. Finally, we performed cell-type deconvolution on 32,078 genes and 3532 spatial spots.

We used the scRNA-seq data from an IPF lung in a previous study as the reference [44]. This dataset consists of 60,651 genes and 12,070 cells. These cells have already been annotated into 39 cell types. Some of the cell types had insufficient cells to provide sufficient information to perform deconvolution. We reannotated the scRNA-seq dataset with 44 cell types in total and selected major cell types with a sufficient number of cells. Therefore, we only considered 11,227 cells from 26 major cell types, and we further filtered out the genes not expressed on these cells. Finally, a final set of 35,483 genes and 11,227 cells serves as the reference scRNA-seq data for deconvolution.

We choose the top 2000 highly variable genes and a set of manually selected 2534 marker genes, corresponding to 3101 unique genes for the CVAE component in SDePER. The number of cells per pseudo-spot is set to be 2–10 cells as provided by expert advice; 1788 cell-type marker genes are used in GLRM component. The hyperparameter ${\lambda }_{r}$ is chosen to be 0.72 and ${\lambda }_{l}$ to be 13.895. The running time of SDePER is 8.58 h in total using a 64-core, 100 GB RAM, Intel Xeon 2.6 GHz CPU machine.

## Supplementary Information

Supplementary Material 1.

Supplementary Material 2.

## Data Availability

The SDePER implementation is freely available at Github (https://github.com/az7jh2/SDePER) [69] and Zenodo (https://zenodo.org/doi/10.5281/zenodo.8328020) [70]. The source code is released under MIT license. A Docker image of SDePER is also freely available at Docker Hub (https://hub.docker.com/r/az7jh2/sdeper) [71]. The scripts used to conduct all the simulation and real data analyses are freely available at Github (https://github.com/az7jh2/SDePER_Analysis) [72] and Zenodo (https://zenodo.org/doi/10.5281/zenodo.13702536) [73] together with all the simulated data and real data. This study assembles five publicly available datasets and one private dataset generated in the laboratory of Dr. Naftali Kaminski. The public datasets used in the simulation studies include the STARmap data (https://kangaroo-goby.squarespace.com/data) and inDrops data (GSE102827) [74] with the external reference data (GSE115746) [75]. The three public datasets used in the real data analyses include the MOB data (https://www.spatialresearch.org/resources-published-datasets/doi-10-1126science-aaf2403/) [76] with its reference data (GSE121891) [77], melanoma data (https://www.spatialresearch.org/resources-published-datasets/doi-10-1158-0008-5472-can-18-0747/) with its reference data (GSE115978) [78], and breast cancer data [79] with its reference data (GSE176078) [80]. The private dataset includes the IPF data (GSE231385) [81] and its reference scRNA-seq data (GSE136831) [82].
