# Supplementary material for: SDePER: a hybrid machine learning and regression method for cell-type deconvolution of spatial barcoding-based transcriptomic data
Source: Genome Biol. 2024 Oct 14;25:271. doi: 10.1186/s13059-024-03416-2 (PMC11475911; doi:10.1186/s13059-024-03416-2)
Supplement: Supplementary file 1 — Supplementary Material 1. [file 13059_2024_3416_MOESM1_ESM.docx]

**Supplementary Information**

**SDePER: A hybrid machine learning and regression method for cell type deconvolution of spatial barcoding-based transcriptomic data**

Yunqing Liu^1,10^, Ningshan Li^1,2,3,10^, Ji Qi^1^, Gang Xu^1,4^, Jiayi Zhao^1^, Nating Wang^1^, Xiayuan Huang^1^, Wenhao Jiang^1^, Huanhuan Wei^1,5^, Aurélien Justet^5,6^, Taylor S. Adams^5^, Robert Homer^7^, Amei Amei^4^, Ivan O. Rosas^8^, Naftali Kaminski^5^, Zuoheng Wang^1,*^, Xiting Yan^5,1,*^

^1^Department of Biostatistics, Yale School of Public Health, New Haven, CT, USA

^2^SJTU-Yale Join Center for Biostatistics and Data Science, Department of Bioinformatics and Biostatistics, School of Life Sciences and Biotechnology, Shanghai Jiao Tong University, Shanghai, China

^3^The Second Affiliated Hopistal of The Chinese University of Hong Kong, Shenzhen, Shenzhen, Guangdong, China

^4^Department of Mathematical Sciences, University of Nevada, Las Vegas, NV, USA

^5^Section of Pulmonary, Critical Care and Sleep Medicine, Yale School of Medicine, New Haven, CT, USA

^6^Service de Pneumologie, Centre de Competences de Maladies Pulmonaires Rares, CHU de Caen UNICAEN, CEA, CNRS, ISTCT/CERVOxy Group, GIP CYCERON, Normandie University, Caen, France

^7^Department of Pathology, Yale School of Medicine, New Haven, CT, USA

^8^Department of Medicine, Baylor College of Medicine, Houston, TX, USA

^9^Department of Biomedical Informatics & Data Science, Yale School of Medicine, New Haven, CT, USA

^10^These authors contributed equally

^*^Corresponding author

Correspondence: zuoheng.wang@yale.edu ; xiting.yan@yale.edu

**Supplementary Figures**

**Fig. S1. Correlation between predicted cell type proportion and ground-truth in simulated data.** For each method and each type of reference data (external or internal), the correlation matrix between predicted cell type proportion and underlying true proportion is shown.

**Fig. S2. Comparison of the accuracy in estimated cell type proportions.** The true and predicted cell type proportion of (A) L4 excitatory neurons, (B) L5 excitatory neurons, (C) L6 excitatory neurons, and (D) oligodendrocytes by each method across all simulated spots are shown.

**Fig. S3.** **Performance evaluation and comparison in sequencing-based simulation study.** Boxplots show the median (center line), interquartile range (hinges), and 1.5 times the interquartile (whiskers) of RMSE, JSD, Pearson’s correlation and FDR using external scRNA-seq reference and internal single-cell level spatial reference.

**Fig. S4. Performance evaluation and comparison in high density sequencing-based simulation study.** Boxplots show the median (center line), interquartile range (hinges), and 1.5 times the interquartile (whiskers) of RMSE, JSD, Pearson’s correlation and FDR using external scRNA-seq reference and internal single-cell level spatial reference. The X-axis label indicates the cell density in a single spot. Compared to ‘1x’ (the sequencing-based simulated data), the ‘3x’ and ‘6x’ settings have 3 and 6 times the number of cells per spot, respectively.

**Fig. S5. Performance evaluation and comparison in Ablation test on SDePER components in STARmap-based simulation study.** Boxplots show the median (center line), interquartile range (hinges), and 1.5 times the interquartile (whiskers) of RMSE, JSD, Pearson’s correlation and FDR using external scRNA-seq reference and internal single-cell level spatial reference. “NO PlatEffRmv” means conducting cell type deconvolution while disregarding platform effect, meaning that neither CVAE nor an additive gene-wise platform effect term is utilized. “NO pseudo spots” means training the CVAE without incorporating pseudo-spots in the training data. “NO LASSO” and “NO Laplacian” means fitting the graph Laplacian regularized model without the sparsity penalty and the spatial correlation constraint, respectively.

**Fig. S6. Performance evaluation and comparison in Ablation test on adaptive Lasso in STARmap-based simulation study with a subset of 5 cell types.** Boxplots show the median (center line), interquartile range (hinges), and 1.5 times the interquartile (whiskers) of RMSE, JSD, Pearson’s correlation and FDR using external scRNA-seq reference and internal single-cell level spatial reference. “NO LASSO” means fitting the graph Laplacian regularized model without the sparsity.

**
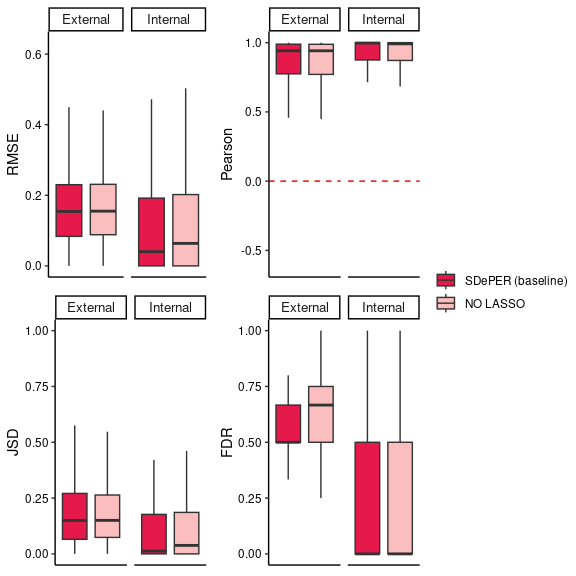
**

**Fig. S7. Performance evaluation and comparison in Ablation test on SDePER components in sequencing-based simulation study.** Boxplots show the median (center line), interquartile range (hinges), and 1.5 times the interquartile (whiskers) of RMSE, JSD, Pearson’s correlation and FDR using external scRNA-seq reference and internal single-cell level spatial reference. The X-axis label indicates the cell density in a single spot. Compared to ‘1x’ (the sequencing-based simulated data), the ‘3x’ and ‘6x’ settings have 3 and 6 times the number of cells per spot, respectively. “NO PlatEffRmv” means conducting cell type deconvolution while disregarding platform effect, meaning that neither CVAE nor an additive gene-wise platform effect term is utilized. “NO pseudo spots” means training the CVAE without incorporating pseudo-spots in the training data. “NO LASSO” and “NO Laplacian” means fitting the graph Laplacian regularized model without the sparsity penalty and the spatial correlation constraint, respectively.

**Fig. S8. Performance of Oligo cell type deconvolution in settings with down sampled Oligo cells in reference for the STARmap-based simulation.** The X-axis represents the number of Oligo cells in the reference set used for cell type deconvolution. "all" on the X-axis indicates that all Oligo cells are included in the analysis (278 Oligo cells for the internal reference and 61 for the external reference). The Root Mean Squared Error (RMSE) for Oligo in a single spot is equivalent to the absolute difference between the estimated and true Oligo proportions. The False Negative Rate (FNR) is calculated as the percentage of spots where Oligo cells were not detected, and the False Discovery Rate (FDR) is calculated as the percentage of spots where Oligo cells were falsely identified.

**Fig. S9. Performance of Oligo cell type deconvolution in spots containing 3 to 6 total cells in the STARmap-based simulation**. The X-axis represents the number of Oligo cells per spot, followed by their proportions within those spots. The Relative Absolute Error (RAE) for Oligo in a single spot is calculated as the absolute difference between the estimated and true Oligo proportions, divided by the true proportion in that spot. The False Negative Rate (FNR) is calculated as the percentage of spots where Oligo cells were not detected.

**Fig. S10. UMAP of the scRNA-seq dataset from mouse olfactory bulb**

**Fig. S11. Expression map of marker genes of GC, M/TC, PGC, OSNs, EPL-IN in the MOB dataset.** Comparison of these heatmaps to Fig. 3A confirms the dominance of each cell type in the corresponding annotated layer based on the histology staining.

**Fig. S12. Comparison of the predicted and imputed cell type proportion at different resolution levels between SDePER and CARD in the MOB dataset.** The original resolution was 200 $\mu m$ which correspond to the estimated cell type proportions from the deconvolution. The three higher resolution levels included 160, 114 and 80$\mu m$, for which the cell type proportions were imputed based on the estimated cell type proportions at the original resolution (200 $\mu m$).


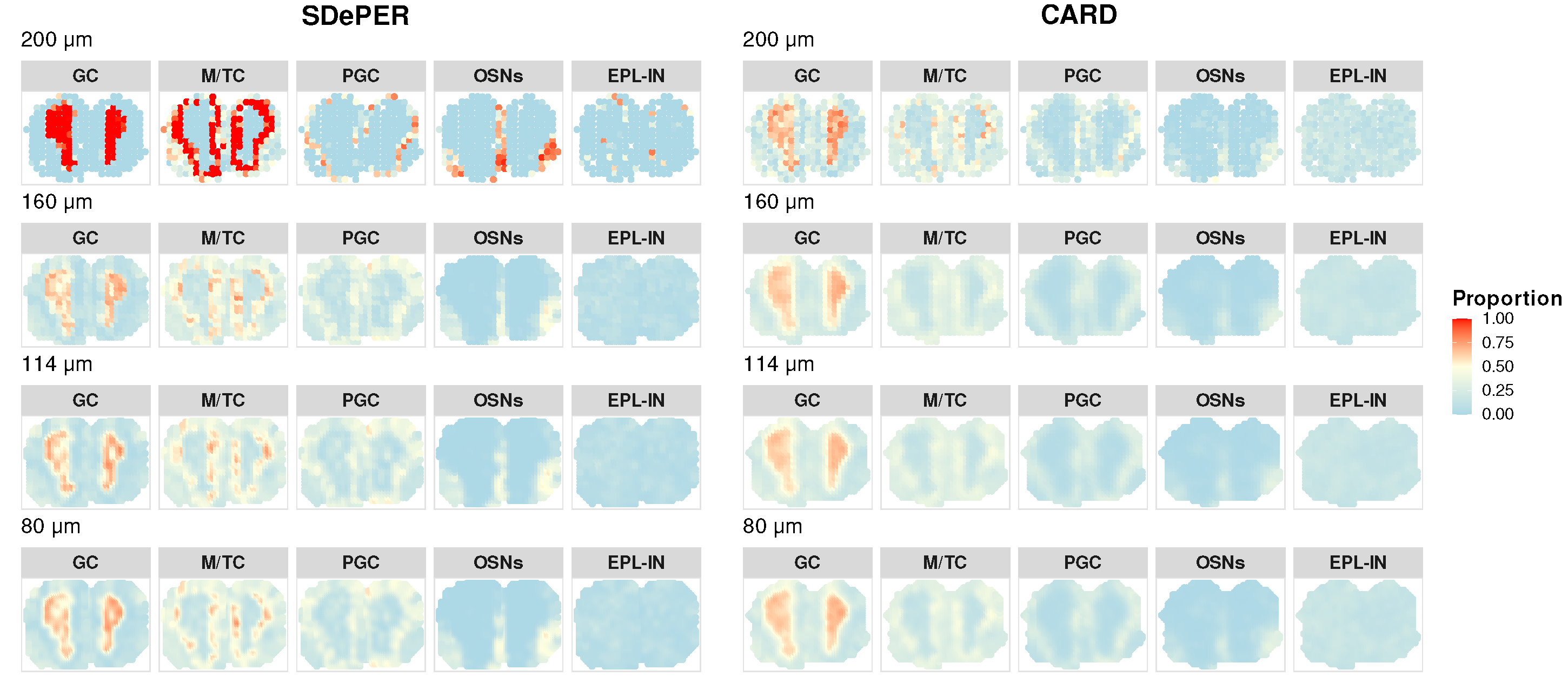


**Fig. S13. Visualization of the imputed expression of layer marker genes at different resolution levels by SDePER and CARD in the MOB dataset.** Three enhanced resolution levels included 160, 114 and 80$\mu m$, for which the expression of each gene was imputed based on the predicted cell type proportions and the ST data at the original resolution (200 $\mu m$).

**Fig. S14. Visualization of expression of cell type marker genes in the melanoma dataset.** One marker was chosen per cell type and demonstrated from left to right and top to bottom for malignant, CAF, macrophage, B cell, T cell, NK cell, endothelial in melanoma spatial dataset, respectively.

**Fig. S15. UMAP of the scRNA-seq dataset from untreated metastatic melanoma samples from human lymph nodes.**

**Fig. S16. Comparison of the predicted and imputed cell type proportion at different resolution levels between SDePER and CARD in the melanoma dastaset.** The original resolution was 200 $\mu m$ which correspond to the estimated cell type proportions from the deconvolution. The three higher resolution levels included 160, 114 and 80$\mu m$, for which the cell type proportions were imputed based on the estimated cell type proportions at the original resolution (200 $\mu m$).


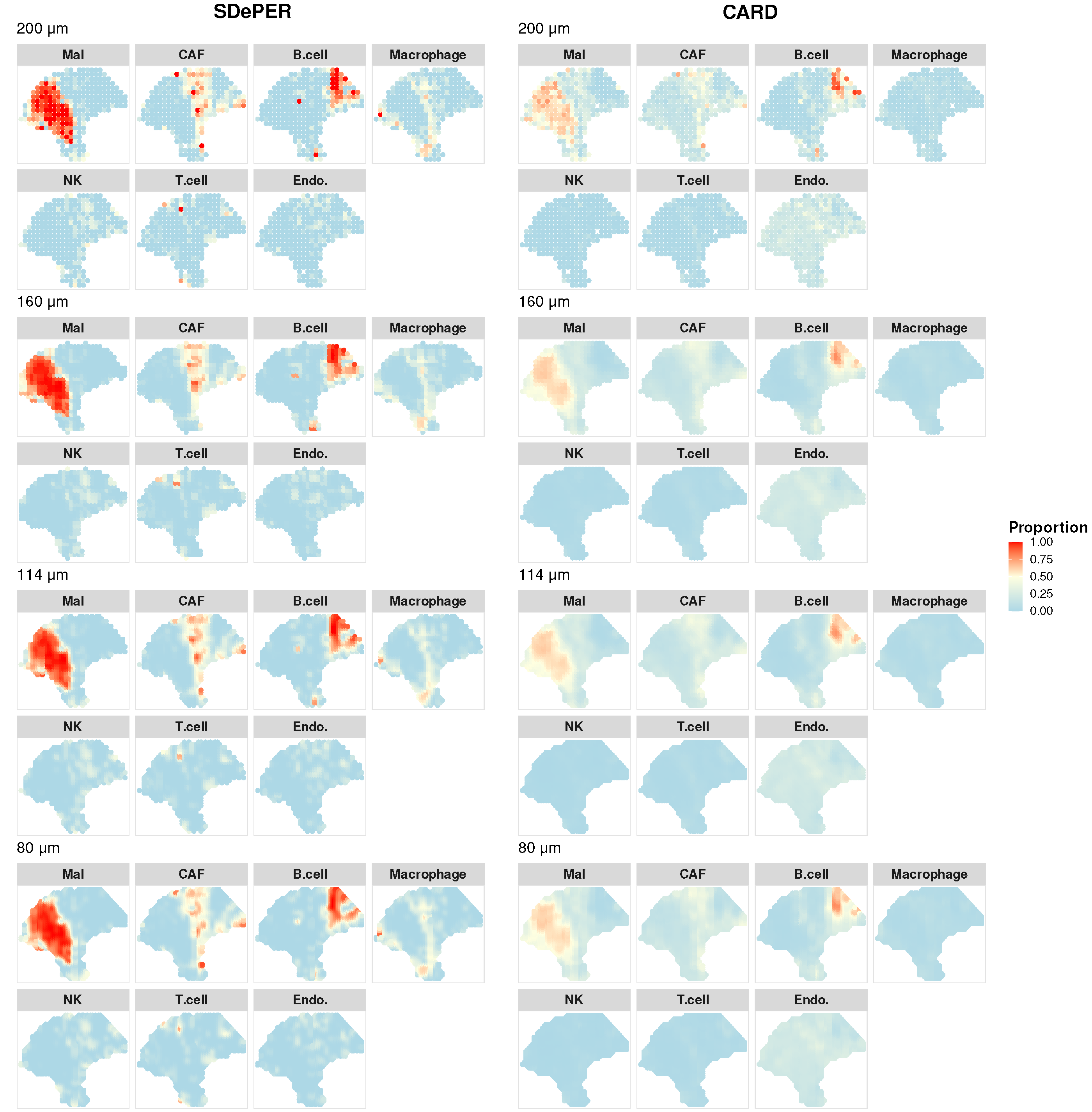


**Fig. S17. Visualization of the imputed expression of layer marker genes at different resolution levels by SDePER and CARD in the melanoma dataset.** Three enhanced resolution levels included 160, 114 and 80$\mu m$, for which the expression of each gene was imputed based on the predicted cell type proportions and the ST data at the original resolution (200 $\mu m$).

**Fig. S18. Expression map of marker genes for different cell types in the breast cancer dataset.** One marker was chosen per cell type and demonstrated from left to right and top to bottom for cancer epithelial, CAF, plasma, myeloid, PVL, endothelial, B cell, T cell, normal epithelial, respectively.

**Fig. S19. UMAP of the scRNA-seq dataset from 5 HER2-positive patients.**

**Fig. S20. Comparison of the predicted and imputed cell type proportion at different resolution levels between SDePER and CARD in the breast cancer dataset.** The original resolution was 200 $\mu m$ which correspond to the estimated cell type proportions from the deconvolution. The three higher resolution levels included 160, 114 and 80$\mu m$, for which the cell type proportions were imputed based on the estimated cell type proportions at the original resolution (200 $\mu m$).


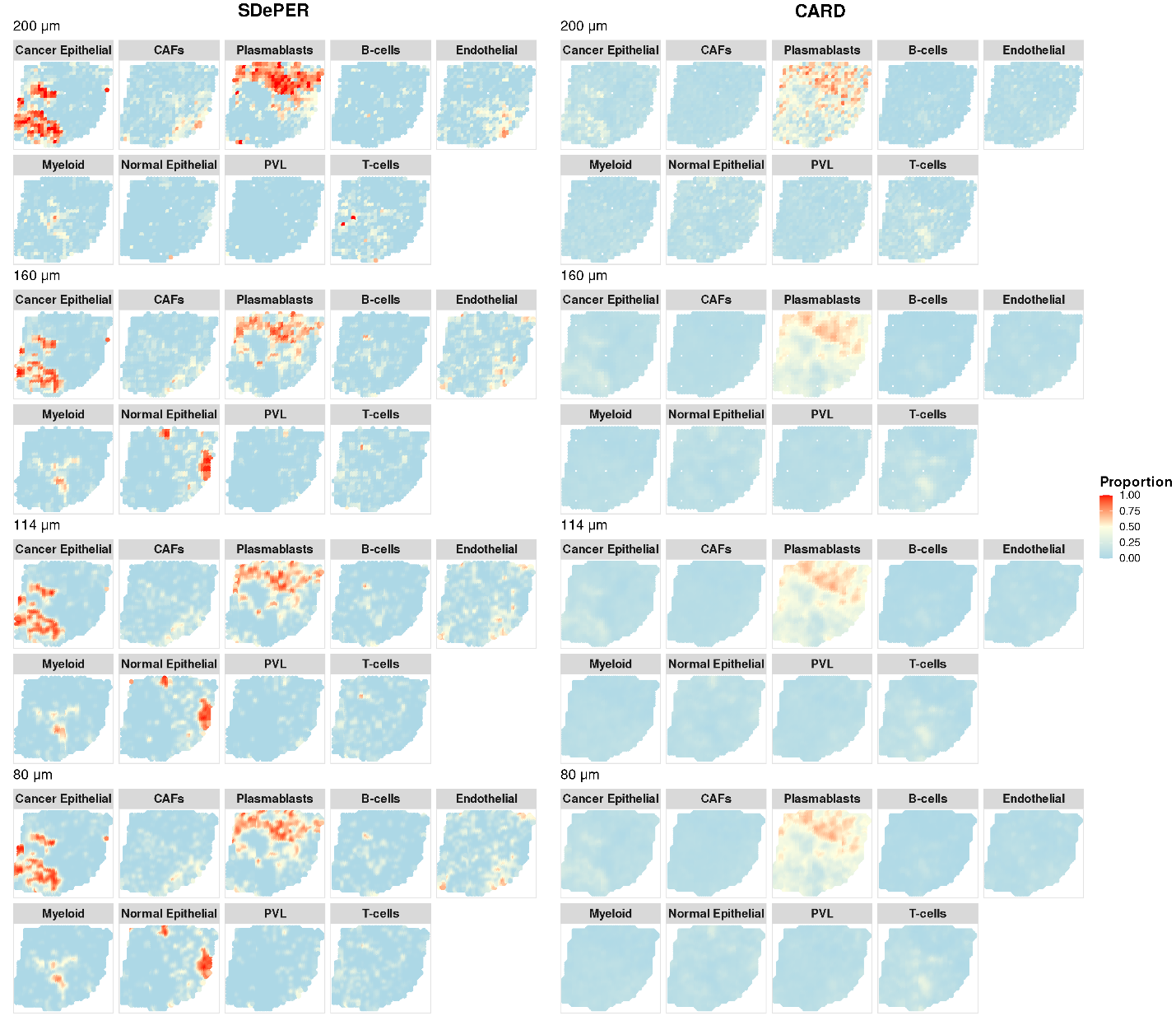


**Fig. S21. Visualization of the imputed expression of layer marker genes at different resolution levels by SDePER and CARD in the breast cancer dataset.** Three enhanced resolution levels included 160, 114 and 80$\mu m$, for which the expression of each gene was imputed based on the predicted cell type proportions and the ST data at the original resolution (200 $\mu m$).

**Fig. S22. Visualization of the co-localization of B cell, T cell and Myeloid cells in the breast cancer dataset**. Color of each spot shows the estimated cell type proportion of the corresponding cell type. Spots with red circle are the TSL region spots shown in the original publication.

**Fig. S23. UMAP of the scRNA-seq dataset of IPF distal lung parenchyma sample.**

**Fig. S24. Visualization of the predicted cell type proportions by all methods in the IPF dataset.** Four cell types, including AT1, AT2, SMC and Ciliated cells, were demonstrated from top to bottom, respectively.

**Fig. S25. Visualization of the predicted cell type proportions by the SDePER in the IPF dataset**. Eight cell types, including AT1, AT2, Basal, Aberrant Basaloid cells, SMC, Advential Fibroblast, Ciliated cells and Airway Fibroblast, were demonstrated from top to the bottom, respectively.

**Fig. S26. Running time of two components of SDePER on IPF dataset with varying number of genes included in CVAE and GLRM.** A machine with a 64-core 2.6 GHz Intel Xeon CPU and 100 GB of RAM is utilized for conducting speed tests**.** CVAE, conditional variational autoencoder; GLRM, graph Laplacian regularized model.

**
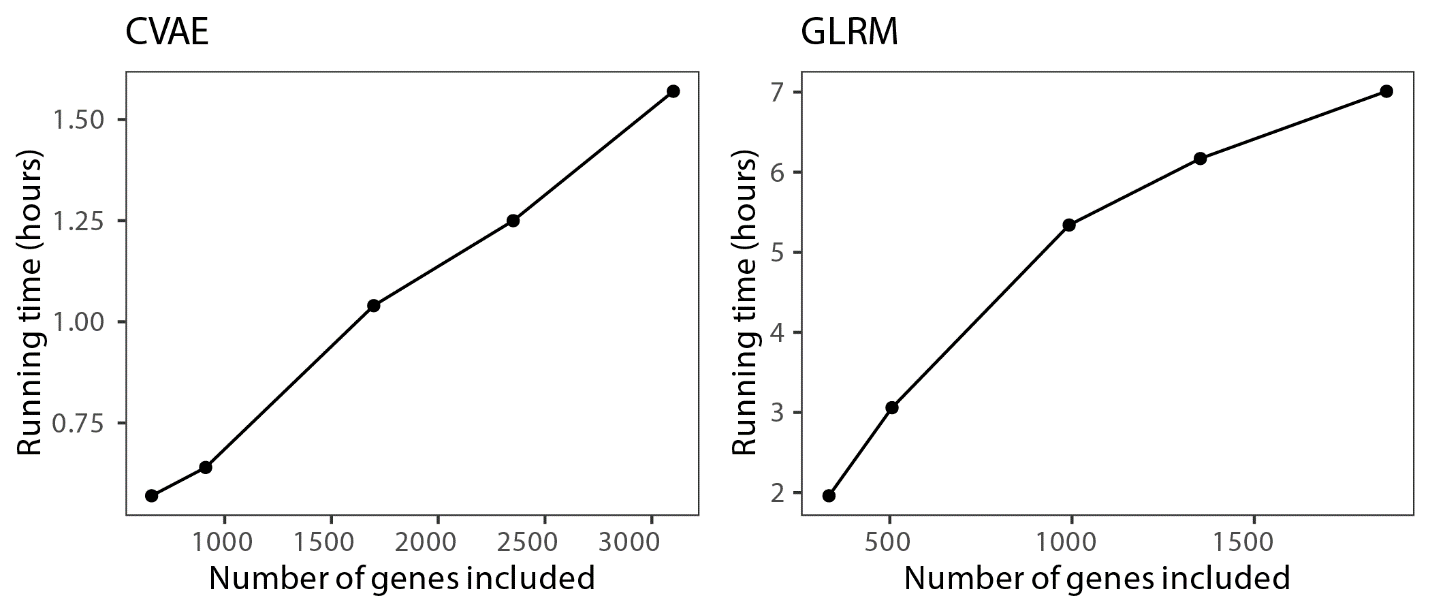
**

**Fig. S27. UMAP of expression of cell type marker genes of spatial spots and scRNA-seq cells after CVAE process in sequencing-based simulation.** External reference was used for cell type deconvolution.

**Fig. S28. Scatter plot of log(mean) versus log(variance) of expressions of cell type marker genes before and after the CVAE process in four real datasets.**
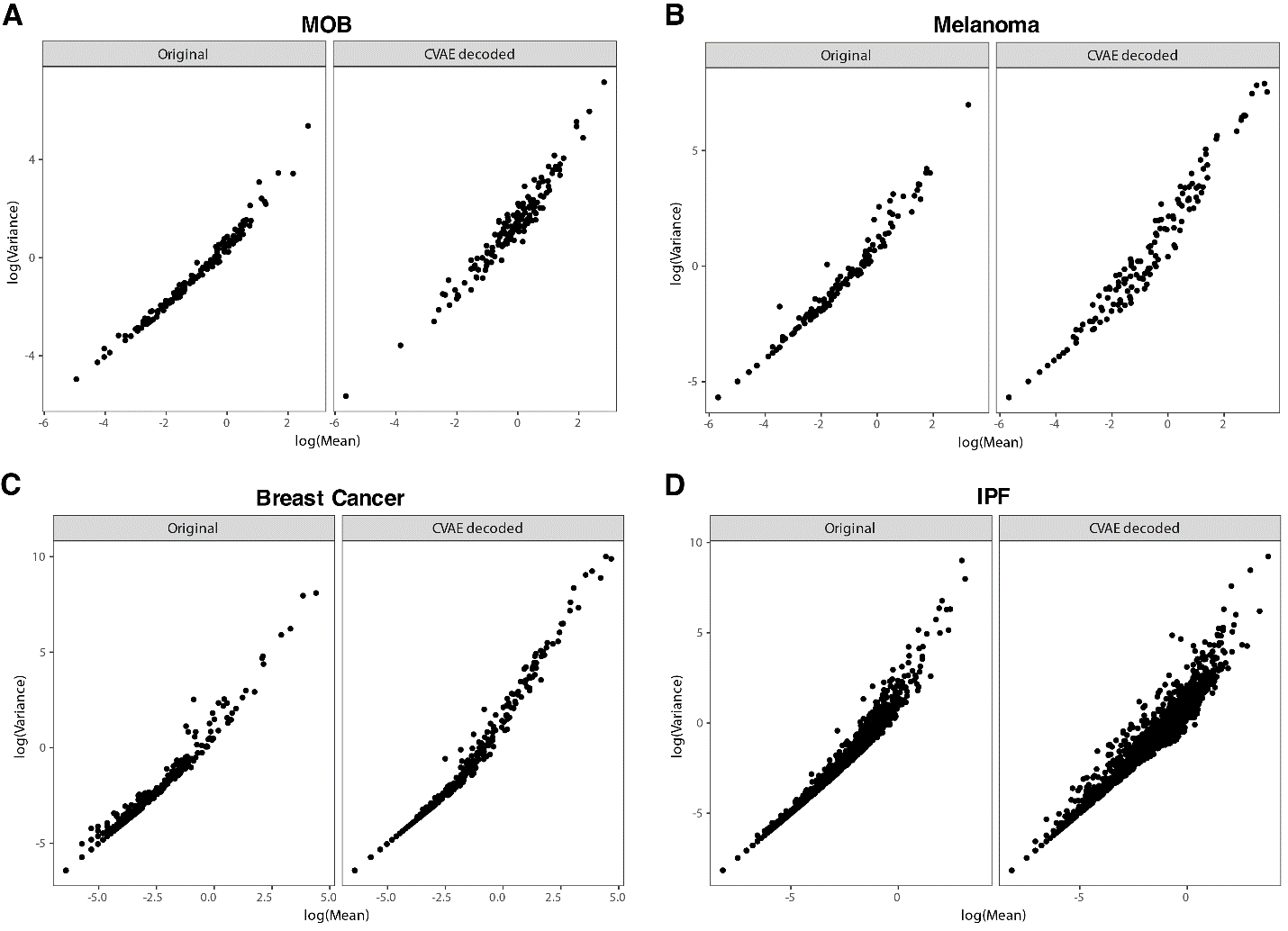


**Fig. S29. Performance of GLRM, Seurat+GLRM and SDePER evaluated using the STARmap-based simulated data with external reference.** Four criteria were used, including Root mean square error (RMSE), Pearson correlation, Jensen-Shannon Divergence (JSD) and false discovery rate (FDR).

**Supplementary Tables**

**Table S1. Quantitative comparison of the accuracy in estimated cell type proportions.** Results for internal and external reference were shown. The median RMSE, JSD, correlation coefficient and FDR was used for the comparison.

| **Methods** | **Reference** | **median_RMSE** | **median_JSD** | **median_cor** | **median_FDR** |
| --- | --- | --- | --- | --- | --- |
| **SDePER** | Internal | 0.101 | 0.179 | 0.892 | 0.500 |
|  | External | 0.120 | 0.274 | 0.819 | 0.667 |
| **GLRM** | Internal | 0.067 | 0.097 | 0.948 | 0.500 |
|  | External | 0.196 | 0.560 | 0.299 | 0.818 |
| **RCTD** | Internal | 0.068 | 0.107 | 0.953 | 0.833 |
|  | External | 0.153 | 0.409 | 0.749 | 0.833 |
| **SpatialDWLS** | Internal | 0.074 | 0.105 | 0.945 | 0.500 |
|  | External | 0.172 | 0.399 | 0.587 | 0.714 |
| **cell2location** | Internal | 0.114 | 0.241 | 0.833 | 0.833 |
|  | External | 0.171 | 0.478 | 0.631 | 0.833 |
| **SONAR** | Internal | 0.062 | 0.092 | 0.958 | 0.833 |
|  | External | 0.171 | 0.468 | 0.645 | 0.833 |
| **SPOTlight** | Internal | 0.148 | 0.383 | 0.842 | 0.818 |
|  | External | 0.174 | 0.490 | 0.667 | 0.818 |
| **CARD** | Internal | 0.108 | 0.220 | 0.855 | 0.833 |
|  | External | 0.194 | 0.511 | 0.286 | 0.833 |
| **DestVI** | Internal | 0.197 | 0.589 | 0.303 | 0.833 |
|  | External | 0.197 | 0.621 | 0.238 | 0.833 |
