# Supplementary material for: SDePER: a hybrid machine learning and regression method for cell-type deconvolution of spatial barcoding-based transcriptomic data
Source: Genome Biol. 2024 Oct 14;25:271. doi: 10.1186/s13059-024-03416-2 (PMC11475911; doi:10.1186/s13059-024-03416-2)
Supplement: Supplementary file 2 — Supplementary Material 2. [file 13059_2024_3416_MOESM2_ESM.docx]

Review History

**First round of review**

**Reviewer 1**

**Were you able to assess all statistics in the manuscript, including the appropriateness of statistical tests used?**

Yes: Yes, the authors posted their analysis codes to GitHub.

**Were you able to directly test the methods?**

Yes

**Comments to author:**

| \| In this manuscript, the authors developed SDePER, a cell type deconvolution method for spatial transcriptomics data. The authors compared SDePER with multiple state-of-the-art methods, including SpatialDWLS, cell2location, SPOTlight, CARD, DestVI, and RCTD. In simulations, they quantitatively evaluated the accuracy of cell type proportion estimation results from different methods using RMSE, Pearson's correlation, JSD, and FDR. In real data analysis, they assessed the performance of various methods in downstream tasks such as spatial region detection and marker gene expression enrichment analysis. SDePER is of particular interest to the spatial transcriptomics research community, especially due to its unique feature of simultaneously addressing three important aspects: the batch effect between ST data and single-cell reference data; the limited number of cell types in each spot; and the spatial correlation of cell type compositions between spots. However, there are major concerns that need to be addressed.  1. In the breast cancer data, the region annotated by the pathologist as an immune infiltrate was identified as a TLS (Tertiary Lymphoid Structure) region in the original data paper. Biologically, this region should predominantly consist of B cells and T cells. However, SDePER missed this important feature. Additionally, the analysis results in other datasets only reaffirmed findings previously reported in the literature, such as regional marker genes, and did not provide novel biological findings or insights. 2. On lines 150-151 of page 8, the authors noted that CARD did not accurately identify the ONL. However, in Figure 3 of CARD's original paper, there is a clear pattern of dominant OSNs in the ONL layer. Given that both SDePER and CARD are using the same spatial transcriptomics MOB dataset and single-cell RNA-seq reference in this example, it would be helpful to double check all methods and verify these results. 3. SDePER's computational efficiency and scalability also raise concerns. As described in the manuscript, it takes 2.58 hours for SDePER to run a small breast cancer data with 613 spots and 109.27 hours to run the lung Visium data with 4992 spots. Given the emergence of large-scale spatial transcriptomics technologies like Slide-Seq, Slide-Seq V2 and Seq-scope with tens of thousands of locations, the ability of SDePER to handle such extensive datasets efficiently is questionable.  4. In the simulations, the authors used the publicly available STARMap dataset to create synthetic spatial datasets. However, it's important to note that the in situ hybridization-based technique of STARMap differs significantly from the sequencing-based methods of 10X ST (used in breast cancer, melanoma, and mouse OB data) and Visium (used in lung data). This difference could potentially bias the simulated data.  5. Several major concerns regarding the use of CVAE for batch effect removal: a. The authors mention on lines 116-119 of page 7 that using an internal reference without platform effects led to reduced performance in SDePER. How does one balance the introduction of noise in the decoded data with the removal of batch effects? This concern also raises the question of whether important biological signals might be lost during the CVAE process. b. To help readers and users clearly understand this method, it will be helpful to illustrate the differences between CVAE and GLRM in Figure 1 and explicitly write down the loss function of CVAE in the Method section. c. The rationale behind choosing CVAE for batch effect removal needs explanation. Considering other methods like Seurat can also remove batch effects, what are the specific benefits of using CVAE? d. What advantages does generating pseudo-spot data from the reference scRNA-seq data as input for CVAE offer? Since cells in each pseudo-spot are randomly selected, this might not accurately reflect the actual distribution of cell types in each real ST spot, potentially introducing noise in the CVAE process. e. To maintain the count nature of data, the authors rescale values obtained from the decoder of CVAE, multiply by 10,000, and round to the nearest integer. Does this approach preserve the mean-variance relationship inherent in the original count data? Does the resulting data still follow the Poisson distribution assumed in the base model?  Minor: 1. It would be helpful to provide example data in the software documentation website. \| \| --- \| |
| --- | --- |

**Reviewer 2**

**Were you able to assess all statistics in the manuscript, including the appropriateness of statistical tests used?**

Yes: I have assessed the statistis

**Were you able to directly test the methods?**

No

**Comments to author:**

The study by Liu et al. describes a new method, SDePER, which is a reference-based deconvolution method for spatial transcriptomics (ST). The authors account for the technical variation between scRNA-seq and ST datasets, and the spatial correlation of cell type composition between adjacent spots. SDePER includes two independent steps to achieve the above goals: A conditional variational autoencoder (CVAE) with different condition variables for scRNA-seq and ST is built to remove batch effects between the two platforms. A regularized Poisson linear regression model is used to estimate the cell-type composition of the spots from ST. In this step, the authors use the adaptive Lasso and Laplacian regularization to enforce the local sparsity and local consistency of cell type composition.

The field of ST data deconvolution has seen a rapid proliferation of computational methods, and SDePER does not demonstrate sufficient innovation to distinguish itself significantly from published methods.

The downstream analysis primarily focuses on visualizing and simply analyzing the enhancement of gene patterns. However, there is a lack of deeper biological analysis of these results.

More specific comments:

1. There are concerns regarding the comparison with published methods such as RCTD and cell2location. The visualization results for these classical methods appear to be unconvincingly poor, raising questions about the comparative analysis.

2. Why does SDePER apply the min-max scaling procedure to the normalized scRNA-seq and ST gene expression profiles? (line 303-304)

3. Identifiability of the spot-specific parameter α_i (linear 342): From the equation in line 342, the mean of the Poisson distribution can be formulated as
log⁡(λ_ij )=α_i+log⁡〖(∑_k▒〖θ_ik μ_kj 〗)+ε_ij 〗,
λ_ij=e^(α_i )⋅e^(ε_ij )⋅(∑_k▒〖θ_ik μ_kj 〗),
N_i λ_ij=N_i⋅e^(α_i )⋅e^(ε_ij )⋅(∑_k▒〖θ_ik μ_kj 〗),
where N_i is a spot-specific total of UMI counts. Why should we consider another spot-specific parameter α_i? And is the spot-specific effect considered by α_i already included by in N_i?

4. What are the results of using regularized Poisson regression alone (without the batch effects removal step)? A further ablation test to verify the necessity for batch effects removal will be more convincing.

5. Why does SDePER use the Poisson distribution instead of the more common negative binomial distribution used in scRAN-seq and ST, which accounts for the over-dispersion property of sequencing data?

6. A similar algorithm SONAR, which also uses Poisson regression to deconvolve ST, has recently been published. What is the difference between SDePER and SONAR, apart from batch effect removal? The comparison results of these two are very similar methods, which should be compared.

7.In Line 355, how to choose the weight qik for adaptive Lasso regularization? In Line 360, I think the adjacency matrix A only characterizes whether there is a connection between two spots. Many articles have defined neighbors of the spot through Euclidean distance. Could the proposed method address the similarity of cell type composition with the spot and its neighbors?

8. The conditional variables in CVAE are label information for some image datasets. In Figure 1, how to define or choose the conditional variables for real datasets in CVAE?

9. More detailed explanations and computational formulas for metrics, such as RMSE, JSD, FDR, and Pearson correlation may be needed.

**Reviewer 3**

**Were you able to assess all statistics in the manuscript, including the appropriateness of statistical tests used?**

Yes: Some related comments are included in my report.

**Were you able to directly test the methods?**

No

**Comments to author:**

In the manuscript entitled "A hybrid machine learning and regression method for cell type deconvolution of spatial barcoding-based transcriptomic data", Liu et al. present an approach for cell type deconvolution of spatial transcriptomic (ST) data using an scRNAseq reference. Their approach consists of a conditional variational autoencoder (CVAE) for transforming the ST spot data and single-cell RNAseq expression counts into the same space, and a graph Laplacian regularized regression model which accounts for cell-type sparsity at a given location (through lasso regularization) as well as the similarity in cell-type composition between spatially neighboring positions (using a Gaussian kernel). They validate their approach by comparison with 6 state-of-the-art methods using 1 simulated dataset (Fig. 2, Supp Fig. S1-S2), and 3 existing real datasets (Fig. 3-5, Supp Fig. S3-S11). Further, they use their model to identify 4 cell-types in a newly generated human IPF dataset (Fig. 6, Supp. Fig S12).

The novelty in this method lies in the consideration of platform effects, sparseness of cell types in a spot, and the correlation between neighboring spots. While these may contribute to better deconvolution results, more evidence is needed to support the effectiveness and necessity of such a framework. Please see some comments and suggestions below.

1. To generate simulated data from the single cell resolution STARmap data, the authors choose a fixed spot resolution (average of 3.6 cells per spot). However, the real datasets in the manuscript have a varying number 5-70 cells per spot. To prove the effectiveness of their approach, more extensive simulation analyses are needed by simulating spots with varying spot-resolutions to account for varying ST spot size (ie. the number of cells in each spot).

2. Real datasets have "vague" and "rare" cell types (Fig. 5) in the ST spot data. The claim that the approach can handle tissue samples with "rare cell types" also needs simulation based evaluation. For example, the authors could consider varying the minimum number of cells of a cell-type required to be present (in scRNAseq reference and simulated ST data) for a cell to be correctly deconvolved.

3. For the real datasets in Fig. 3-4, the authors annotate their spatial data with 4 very broad cell-types (Fig. 3a, Fig. 4a). Could the authors clarify how they annotated the spatial data to obtain the patterns shown in Fig. 3a and Fig. 4a used as groundtruth?

4. Could the authors include visualizations of the reference scRNA-seq datasets for all of the analyses to improve readability? These can be UMAP visualizations with annotated cell types.

5. The pseudo-spot data obtained from scRNA-seq data was used in the model but not used in a supervised learning manner. To show that including such data is beneficial, an ablation test needs to be included to compare results using and not using the pseudo-spot data.

6. To show the necessity of using the regularization to enforce the two constraints: sparseness of cell types in spots and correlation between neighboring spots, ablation tests are needed. In particular, in Fig. 3 and 4, a small number of cell types are considered. In such cases, will the sparseness constraint still be useful?

7. More details on hyperparameter tuning is needed for users to be able to set hyperparameters when using this tool. In particular, how are hyperparameters for the CVAE model chosen for each dataset? In the paragraph from line 417-422: what is the range of parameter search? How to use simulated data to determine best parameters?

8. For Fig. 2B and 2D, please explain how many data points are there in each box and what each point corresponds to.

Some minor comments:

Line 65: Despite the success (remove "of")
Line 66: Address"es" them simultaneously
Line 70: "and varying"
Line 72: "were shown in a previous"
Line 96: "removes" is a strong claim that is not sufficiently justified. Consider "corrects for"
Line 110: "using external reference" consider adding "an" or "the"
Consider swapping Fig. S1 and S2 order since S2 is mentioned before S1 in text
Line 138: "on real data"
Line 143: "composed of one cell type"
Fig. 3B-C: Could the authors comment on Spotlight as well since the results look quite poor
Line 219: It is not obvious how Fig. 5F demonstrates by "visualization" and Fig. 5G by "quantitative measure".
Line 400: Could the authors explain this criteria ? How do they account for biological holes in tissue slices ?
Fig. 3: Why do the authors choose different cell-type specific genes in Fig 3a and 3e ?
Fig. 6C. Incorrect legend. Fig. 6C is not a heatmap.
Fig. S3 is the same as 3A markers with the additional EPL-IN population which is not in 3A. Could the authors add this annotation /marker to 3A ?

**Authors Response**

**Point-by-point responses to the reviewers’ comments:**

We thank the Editors and referees for their thoughtful and constructive comments. Below we detail how each of the comments has been addressed. The reviewers’ comments are in bold and our responses are provided under each comment.

**Reviewer #1:**

**1.      In the breast cancer data, the region annotated by the pathologist as an immune infiltrate was identified as a TLS (Tertiary Lymphoid Structure) region in the original data paper. Biologically, this region should predominantly consist of B cells and T cells. However, SDePER missed this important feature. Additionally, the analysis results in other datasets only reaffirmed findings previously reported in the literature, such as regional marker genes, and did not provide novel biological findings or insights.**

*Response:* We thank reviewer 1 for the insightful comments. For the TLS regions, in the paper publishing the breast cancer data, these regions were predicted by the co-localization of B and T cells but “TLSs are not exclusively inhabited by B and T cells” as described in the paper. In fact, Supplementary Figure 24 in the breast cancer ST data paper (<https://static-content.springer.com/esm/art%3A10.1038%2Fs41467-021-26271-2/MediaObjects/41467_2021_26271_MOESM1_ESM.pdf> ) performed IHC staining of B cells and T cells in the TLS regions. The staining results showed that although B cells are present in the TLS regions, the number of B cells in TLS regions is low compared to the other cells, indicating a low proportion of B cells in the TLS regions.

These TLS regions did not show up in our results (Fig. 5B) because Fig. 5B only showed the dominant cell type for each spot. We checked the co-localization of B and T cells by visualizing the predicted B and T cell proportion by SDePER across spots on the tissue slice (Fig. S27). The results showed that SDePER results were also able to demonstrate the TLS regions, especially when counting co-localization of B cells, T cells and Myeloid cells. Our findings on accumulation of Myeloid cells in the TLS regions is supported by previous literatures [1-3]. We added these results to Results section (page 14 line 292-296).

We compare the analysis results in the real datasets to previous findings reported in the literature to assess the accuracy of all methods. We did not focus on the novel biological findings because this article was submitted as a methodology paper, which should “describe a methodological innovation that is a significant advance over published methods and likely to be of broad utility, but that do not provide significant biological insights”, according to the instructions from the editorial office. In addition, we consider novel biological findings to be results that have been validated by experiments, which is outside the scope of this article.

To accommodate the reviewer’s comment on provide novel biological findings, Furthermore, we examined the results of other important cell types (Fig. S28), including aberrant basaloid cells, adventitial fibroblast and airway fibroblast. Aberrant basaloid cells seem to co-localize well with basal cells. They are also present in the alveoli together with AT I and AT II cells. Adventitial fibroblast was found to co-localize with the vascular smooth muscle cells, suggesting its presence in the vascular compartment. A recent spatial transcriptomic study[4] of IPF lung using the 10x Genomics Xenium platform validated this finding. The airway fibroblasts were found to be present in the vascular compartment instead of in the airway, indicating that a further investigation of the location of these cells in human lungs is needed. These descriptions were added to the Results section (page 16 line 325-332).

**2.      On lines 150-151 of page 8, the authors noted that CARD did not accurately identify the ONL. However, in Figure 3 of CARD's original paper, there is a clear pattern of dominant OSNs in the ONL layer. Given that both SDePER and CARD are using the same spatial transcriptomics MOB dataset and single-cell RNA-seq reference in this example, it would be helpful to double check all methods and verify these results.**

*Response:* When we ran CARD on the MOB dataset, we noticed the difference between our results and Figure 3 of CARD’s original paper. In this revision, we double checked our codes, the tutorial of CARD software as well as the data and codes used in the original CARD paper. We confirmed that the difference was due to the difference in the input data.

The codes that generated Figure 3 in CARD’s original paper can be found here: <https://github.com/YMa-lab/CARD-Analysis/blob/master/Analysis/MOB_CARD.Rmd>. Lines #20 and #30 of the codes show that the reference scRNA-seq data used for the MOB dataset is at: <https://drive.google.com/file/d/1NSkrulMVhDbj9D0WztTofOps7M9ONSyL/view?usp=sharing>. Screenshot of the head of this data can be found in Figure R1, which showed that it is a normalized nUMI data instead of the raw nUMI count data.


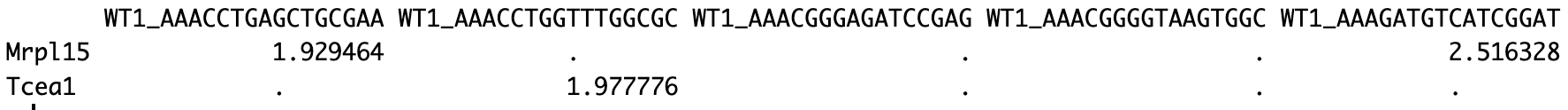


**Figure R1**. Screenshot of the head of the MOB gene expression matrix used to generate results in the Figure 3 of CARD's original paper. The non-integer values suggests that this is the normalized nUMI data instead of the raw nUMI count data.

To confirm that the results in Figure 3 of CARD’s original paper was generated using the normalized nUMI data for the reference scRNA-seq data, we replaced our reference data with this provided normalized data in our codes to apply CARD to the MOB dataset. The results we obtained (Figure R2) successfully replicated CARD’s results in Figure 3 of CARD’s original paper, confirming that results in Figure 3 of CARD’s original paper were generated using the normalized nUMI data for the reference scRNA-seq data instead of the raw nUMI count data.

**Figure R2.** Results of CARD by applying CARD to the normalized data generated by us. The left panel shows the predicted dominant cell type for each spot by CARD. The right panel showed the pie chart of the predicted cell type proportions for each spot. This replicated Figure 3b and 3c of CARD’s results in CARD’s original paper.

When we generated our results of CARD on the MOB dataset, we followed the tutorial of CARD (<https://yma-lab.github.io/CARD/documentation/04_CARD_Example.html>) to use the raw nUMI data instead of the normalized nUMI data for the reference scRNA-seq data. Although we noticed the difference in the results, we chose to follow the tutorial due to the following reasons. First, we need to follow the tutorial to ensure that other researchers can independently reproduce our results. Second, the CARD paper did not detail how they normalized the reference scRNA-seq data for MOB dataset. So we are not able to replicated their normalization. In addition, the reference data of another real dataset (Hippocampus) analyzed in the CARD paper was not normalized so the raw nUMI count data was used as input. These inconsistency in the original paper across different datasets further drove us to follow the tutorial, which should be updated and more mature than the paper.

Taken together, we believe that it is correct to follow the CARD tutorial and use the raw nUMI count in the reference scRNA-seq data as input instead of the normalized nUMI data. Both our codes and the raw nUMI count data we used to generate the results for CARD have been uploaded onto our github repo (<https://github.com/az7jh2/SDePER_Analysis>), which can be found in page 35 line 768.

In addition, as suggested by the reviewer, we also double checked our codes for all other existing methods. We found that some of them have significant updates on the version that we used in our first paper submission. To make sure our comparison results are reproducible and fair, we updated the results of all existing methods throughout this revised manuscript by replacing the old version with the most updated version for all existing methods, including CARD.

**3.      SDePER's computational efficiency and scalability also raise concerns. As described in the manuscript, it takes 2.58 hours for SDePER to run a small breast cancer data with 613 spots and 109.27 hours to run the lung Visium data with 4992 spots. Given the emergence of large-scale spatial transcriptomics technologies like Slide-Seq, Slide-Seq V2 and Seq-scope with tens of thousands of locations, the ability of SDePER to handle such extensive datasets efficiently is questionable.**

*Response:* We improved the computational speed of SDePER by using batch normalization in the CVAE training and setting the initial values of parameters in the GLRM optimization using estimators from the latent space embeddings learned by CVAE. These two changes reduced the computation time to 1.8 hours for the breast cancer dataset and 8.58 hours for the IPF lung Visium dataset. The computational time for each dataset before and after the improvement is listed as follows.

MOB dataset: 0.58 hours ---> 0.56 hours

Melanoma dataset: 0.60 hours ---> 0.56 hours

Breast Cancer dataset: 2.41 hours ---> 1.80 hours

IPF dataset: 109.27 hours ---> 8.58 hours

Based on the model, the computational time of SDePER will linearly increase with the total number of spots. We further evaluated how the number of genes used in the CVAE training and GLRM model fitting affect the computational speed (Fig. S24), which demonstrated a linearly increasing computational time when the number of genes increased. For large-scale spatial transcriptomics data, by selecting limited but representative genes (~500 genes), we could finish the analysis of one 10x Visium tissue slide with about around 3,500 spots in ~2.5 hours. For data with even larger scale, it’s possible to disable the graph Laplacian penalty in SDePER so that it can be run parallelly across different spots. In addition, the computational efficiency of SDePER can be further improved by caching the calculated log-likelihoods in GLRM fitting to avoid repetitive.

We updated the computational time for all datasets in the Results section (line 685, 698, 710 and 748). We also added the above descriptions in the Discussion (page 17 line 348-356)

**4.      In the simulations, the authors used the publicly available STARMap dataset to create synthetic spatial datasets. However, it's important to note that the in situ hybridization-based technique of STARMap differs significantly from the sequencing-based methods of 10X ST (used in breast cancer, melanoma, and mouse OB data) and Visium (used in lung data). This difference could potentially bias the simulated data.**

*Response:* The STARMap technology is a hybrid technology of in situ hybridization and sequencing. The protocol enriches for transcripts using hybridization techniques but the final nUMI is still generated based on sequencing. Therefore, the actual expression levels were assessed using sequencing-based technology, but the targeted genes were enriched using in situ hybridization-based technology.

We agree that the difference between STARMap and 10X ST or Visium may make the results not reflecting many of the real applications because the platform effect might be smaller for cases with both platforms being sequencing-based, especially when both ST data and reference data are from 10x Genomics branded platform. Therefore, we conducted additional simulation study by keeping the physical locations of cells in the STARmap data the same but replacing the gene expression profile of each cell with that of a randomly chosen cell of the same type from an independent scRNA-seq dataset of the same tissue type with matching cell types. The simulation results showed that although a smaller platform effect was observed, SDePER still had better performance compared to the other methods. It achieved the second smallest RMSE (0.088) compared to cell2location (0.082) but had a lower FDR (0.6 vs 0.833). The results were added in the Results (page 7 line 128-133) and in the supplementary materials (Fig. S5).

**5.      Several major concerns regarding the use of CVAE for batch effect removal:
    a.  The authors mention on lines 116-119 of page 7 that using an internal reference without platform effects led to reduced performance in SDePER. How does one balance the introduction of noise in the decoded data with the removal of batch effects? This concern also raises the question of whether important biological signals might be lost during the CVAE process.**

*Response:* We agree that the current version of CVAE might introduce noise to the data, which cause worse performance when there’s no platform effect. However, when the cell density increased by 3 or 6 times, the performance of SDePER was much better than GLRM (Fig. S6) indicating a great performance boost by using CVAE even though it introduces noise, especially when cell density is high.

One potential way to assess the severity of platform effects is to examine the overlap between reference and real ST data in the UMAP before and after the CVAE process. This is similar to the integration analysis which improves overlap between cells of the same type across batches. Larger improvement in the overlap between the two platforms may suggest more severe platform effects. This has been added in Discussion (page 17 line 358-363).

There might be some biological signals lost during the CVAE process. To ensure that this will not bias the results, **first**, we set genes used in the GLRM component to cell type markers after the data transformation by CVAE. In this way, genes that lost signals after the CVAE transformation would be excluded from the regression model. Revisions were made in the Method section to clarify this (page 21 line 447-449). **Second**, to determine whether most of the biological signals were lost by CVAE transformation, we examined whether different cell types in the reference scRNA-seq still separate well after the CVAE transformation. The UMAP of the reference scRNA-seq, pseudo-spots and real spots using the CVAE adjusted data showed good separation between different cell types, indicating that enough biological signals were kept separating different cell types after the CVAE process. The goal of SDePER is to deconvolve the ST data instead of identifying the biological signals. So as long as enough biological signals remained after the CVAE process, SDePER will be able to achieve the goal. We added the UMAPs of CVAE adjusted ST data and reference scRNA-seq data in the sequencing-based simulation into the supplementary material (Fig. S26) and added the clarifications above in the results section (page 20 line 429-430) to demonstrate this.

In the SDePER software provided on the github, we added a function to provide these UMAPs as diagnostic plots for the CVAE training. The tutorial has been updated accordingly.

**b.  To help readers and users clearly understand this method, it will be helpful to illustrate the differences between CVAE and GLRM in Figure 1 and explicitly write down the loss function of CVAE in the Method section.**

*Response:* We revised Figure 1 to highlight and further explain the difference between CVAE and GLRM. The loss function of CVAE was added in the Method section (page 18 line 383-391).

**c.  The rationale behind choosing CVAE for batch effect removal needs explanation. Considering other methods like Seurat can also remove batch effects, what are the specific benefits of using CVAE?**

*Response:* The GLRM component in SDePER regresses the CVAE adjusted real spot data to the cell type-specific expression profiles from the CVAE adjusted reference scRNA-seq data using a generalized linear mixed effects model, which assumes an additive and linear relationship between the ST and reference data. To ensure this model assumption holds, it is critical to keep the biological meaning or the distribution of the data after the adjustment for platform effects removal. Other batch effect adjustment methods, such as Seurat, Harmony and so on, map different batches of data into a common latent space which does not necessarily keep the biological meaning or the data distribution of each gene. So the batch adjusted data by these methods for each gene does not reflect its real gene expression levels. However, CVAE is a deep generative model which learns the true data distribution in a latent space and to generate new data points from the learned distribution. The generated new data will have the original data distribution and therefore keep the original biological meaning. Therefore, the CVAE adjusted data are still gene expression data which guarantees the GLRM model assumption on the linear additive relationship between the ST and reference data. If the adjusted data is not expression data anymore, this assumption cannot hold so GLRM component will fail. We added the clarifications to Method section (page 20 line 433-444).

**d.  What advantages does generating pseudo-spot data from the reference scRNA-seq data as input for CVAE offer? Since cells in each pseudo-spot are randomly selected, this might not accurately reflect the actual distribution of cell types in each real ST spot, potentially introducing noise in the CVAE process.**

*Response:* CVAE assumes a Gaussian distribution in the latent space for both scRNA-seq and ST conditions. So it is critical for the data under both conditions to cover similar spectrum of cell type compositions. If we do not include the pseudo-spot data, all data under the scRNA-seq condition have only one single cell type, which will be extremely different from the real spot data under the ST condition because each spot could have cells from multiple cell types. To match the two data distributions between the two conditions, we generated the pseudo-spot data with randomly chosen cell type compositions and cells from scRNA-seq platform, which expands the spectrum of true cell type compositions of the scRNA-seq condition to match that of the ST condition. We added the explanation into the method section (page 19 line 392-396).

To demonstrate the importance of the pseudo-spot inclusion, we conducted ablation test by running SDePER without any pseudo-spot data. The results (Fig. S7, S8) showed that the including pseudo-spot data is important for SDePER to successfully remove the platform effects. The results were added into the supplementary material as Fig. S7 and S8. Descriptions of the results were added into the Results under the Alation Study (page 8 line 144-160) and in the supplementary materials (Fig. S7, S8).

**e.  To maintain the count nature of data, the authors rescale values obtained from the decoder of CVAE, multiply by 10,000, and round to the nearest integer. Does this approach preserve the mean-variance relationship inherent in the original count data? Does the resulting data still follow the Poisson distribution assumed in the base model?**

*Response:* We checked the mean-variance plot across different genes before and after the CVAE process. The results showed that the mean-variance relationship did not change. In addition, SDePER assumes the data follow a Poisson log-normal distribution which considers data dispersion. The mean-variance relationship for all 4 real datasets also confirms that data dispersion is present, which fit our model assumption. We added this in the supplementary material as Fig. S25 and description of the results in the Method section (page 20 line 431-432, 558-559, page 22 line 459-460).

**Minor:
1. It would be helpful to provide example data in the software documentation website.**

*Response:* We uploaded all simulated datasets and four real datasets we used in our manuscript onto the github repo of SDePER (https://github.com/az7jh2/SDePER_Analysis).

**Reviewer #2**:

1. **There are concerns regarding the comparison with published methods such as RCTD and cell2location. The visualization results for these classical methods appear to be unconvincingly poor, raising questions about the comparative analysis.**

*Response:* We double checked our codes and did not find mistakes. We also double checked the tutorials of all existing methods and followed the instructions on parameter setting. The example data and our codes to run these methods were uploaded onto the SDePER github repo (<https://github.com/az7jh2/SDePER_Analysis>) to ensure reproducibility.

We did find that many of the existing methods have significant updates on the versions that we compared to in our first paper submission. So we updated the results of existing methods throughout this revised manuscript using the most updated version. The performance of cell2location did have significant improvement. However, in the updated results, SDePER still had the best performance. The updated results were added throughout the manuscript in this revision.

1. **Why does SDePER apply the min-max scaling procedure to the normalized scRNA-seq and ST gene expression profiles? (line 303-304)**

*Response:* The training of CVAE is sensitive to the differences in data range across different genes. Therefore, we use the min-max scaling procedure to match the data range across different genes. We added the clarification to the Method section (page 19 line 408-409).

1. **Identifiability of the spot-specific parameter** $\boldsymbol{\alpha}_{\boldsymbol{i}}$ **(linear 342): From the equation in line 342, the mean of the Poisson distribution can be formulated as**

$$\log\left( \boldsymbol{\lambda}_{\boldsymbol{ij}} \right)\boldsymbol{=}\boldsymbol{\alpha}_{\boldsymbol{i}}\boldsymbol{+}\log\left( \boldsymbol{\sum}\boldsymbol{\theta}_{\boldsymbol{ik}}\boldsymbol{\mu}_{\boldsymbol{kj}} \right)\boldsymbol{+}\boldsymbol{\varepsilon}_{\boldsymbol{ij}}\boldsymbol{,}$$

$$\boldsymbol{\lambda}_{\boldsymbol{ij}}\boldsymbol{=}\boldsymbol{e}^{\boldsymbol{\alpha}_{\boldsymbol{i}}}\boldsymbol{\cdot}\boldsymbol{e}^{\boldsymbol{\varepsilon}_{\boldsymbol{ij}}}\boldsymbol{\cdot}\left( \boldsymbol{\sum}\boldsymbol{\theta}_{\boldsymbol{ik}}\boldsymbol{\mu}_{\boldsymbol{kj}} \right)\boldsymbol{,}$$

$$\boldsymbol{N}_{\boldsymbol{i}}\boldsymbol{\lambda}_{\boldsymbol{ij}}\boldsymbol{=}\boldsymbol{N}_{\boldsymbol{i}}\boldsymbol{\cdot}\boldsymbol{e}^{\boldsymbol{\alpha}_{\boldsymbol{i}}}\boldsymbol{\cdot}\boldsymbol{e}^{\boldsymbol{\varepsilon}_{\boldsymbol{ij}}}\boldsymbol{\cdot}\left( \boldsymbol{\sum}\boldsymbol{\theta}_{\boldsymbol{ik}}\boldsymbol{\mu}_{\boldsymbol{kj}} \right)\boldsymbol{,}$$

**where** $\boldsymbol{N}_{\boldsymbol{i}}$ **is a spot-specific total of UMI counts. Why should we consider another spot-specific parameter** $\boldsymbol{\alpha}_{\boldsymbol{i}}$**? And is the spot-specific effect considered by** $\boldsymbol{\alpha}_{\boldsymbol{i}}$ **already included by in** $\boldsymbol{N}_{\boldsymbol{i}}$**?**

*Response:* $N_{i}$ is the sequencing depth calculated as the total number of nUMIs which represents a technical factor. It is a constant. $\alpha_{i}$ is a parameter representing the variation in gene expression across different spatial locations, which will be estimated by the model fitting. We considered $\alpha_{i}$ because previous studies demonstrated that gene expression profile of cells from the same type could vary depending on where they are located in the tissue, potentially due to the influences from neighboring cells or tissue microenvironment. Therefore, $\alpha_{i}$ is different and not included in $N_{i}$. We added the clarification for this in Method (page 22 line 475-478).

1. **What are the results of using regularized Poisson regression alone (without the batch effects removal step)? A further ablation test to verify the necessity for batch effects removal will be more convincing.**

*Response:* We conducted an ablation test on SDePER by disabling the CVAE component and compare the results to SDePER. The result (Fig. S7 and Fig. S8) showed that disabling the CVAE component significantly decreased the performance of SDePER for external reference, i.e. when platform effects exist. It also showed that when cell density is high, disabling CVAE lead to decreased performance of SDePER even when there was zero platform effect for internal reference. These results were added in Results (page 8 line 144-160).

1. **Why does SDePER use the Poisson distribution instead of the more common negative binomial distribution used in scRAN-seq and ST, which accounts for the over-dispersion property of sequencing data?**

*Response:* In our model, the observed nUMI is assumed to follow a Poisson distribution with a mean of $\log\left( \lambda_{ij} \right)=\alpha_{i}+\log\left( \sum\theta_{ik}\mu_{kj} \right)+\varepsilon_{ij}$, where $\varepsilon_{ij}$ follows a Gaussian distribution with a heavy tail (page 22 line 470-475). Therefore, SDePER uses the Poisson-lognormal distribution, which does consider over-dispersion like the negative binomial distribution.

1. **A similar algorithm SONAR, which also uses Poisson regression to deconvolve ST, has recently been published. What is the difference between SDePER and SONAR, apart from batch effect removal? The comparison results of these two are very similar methods, which should be compared.**

*Response:* We thank the reviewer for suggesting SONAR. The differences between SONAR and SDePER apart from batch effect removal are as follows. First, SDePER uses Poisson-Lognormal distribution and SONAR uses Poisson-Gamma distribution. Specifically, SDePER models the log-transformed expression rate in the Poisson distribution as a linear function of cell type-specific expression profiles, while SONAR models the expression rate, instead of the log-transformed expression rate. Second, SONAR does not consider sparsity in the cell type composition while SDePER does using the adaptive LASSO penalty. Third, SONAR considers the spatial correlation using a spatial weighting function on the local likelihood and the weights were decided based on the similarity of expression between neighboring spots. SDePER uses graph Laplacian penalty to consider spatial correlation which can use weighted adjacency matrix to considers physical distances between spots. For simplicity, the results shown in our current manuscript are implemented with a unweighted adjacency matrix which considers whether neighboring spots are close enough or not. We also added SONAR to the list of compared methods throughout the manuscript in this revision.

1. **In Line 355, how to choose the weight qik for adaptive Lasso regularization? In Line 360, I think the adjacency matrix A only characterizes whether there is a connection between two spots. Many articles have defined neighbors of the spot through Euclidean distance. Could the proposed method address the similarity of cell type composition with the spot and its neighbors?**

*Response:* qik was calculated as the reciprocal of the maximum likelihood estimation (MLE) of the proportion of cell type k in spot i from the SDePER without the adaptive LASSO and graph Laplacian penalties (base model). Clarifications were added to Method (page 23 line 484-485).

The adjacency matrix A in SDePER can be set to a weighted adjacency matrix reflecting the Euclidean distance between spots although results in this article were demonstrated using the unweighted adjacency matrix for simplicity. We added this clarification to the Method section (page 23 line 489-490).

1. **The conditional variables in CVAE are label information for some image datasets. In Figure 1, how to define or choose the conditional variables for real datasets in CVAE?**

*Response:* The conditional variable in CVAE is defined to be 0 or 10 by default with 0 representing the scRNA-seq platform and 10 representing the spatial transcriptomic platform. So in the training data, the conditional variable for all the single cells in the reference data and the pseudo-spot data is equal to 0. The conditional variable for all the real spot data has a value of 10. We modified our descriptions on this in the Method section (page 19 line 411-414).

1. **More detailed explanations and computational formulas for metrics, such as RMSE, JSD, FDR, and Pearson correlation may be needed.**

*Response:* We added the formulas for RMSE, JSD, FDR and Pearson correlation in the method section (page 31 line 665-670).

**Reviewer #3**:

**1. To generate simulated data from the single cell resolution STARmap data, the authors choose a fixed spot resolution (average of 3.6 cells per spot). However, the real datasets in the manuscript have a varying number 5-70 cells per spot.  To prove the effectiveness of their approach, more extensive simulation analyses are needed by simulating spots with varying spot-resolutions to account for varying ST spot size (ie. the number of cells in each spot).**

*Response:* As suggested by the reviewer, we conducted simulations for high cell density to generate spots with more cells. To simulate ST data with high cell density, we kept the physical spot size the same as in the sequencing-based simulation but increased the total number of cells in each spot by 3 or 6 times, which correspond to approximately 3 to 36 cells and 6 to 72 cells per spot with an average of 10.8 and 21.6 cells per spot, respectively. In each spot, the cell type proportions remained the same but for each existing cell type, 3 or 6 times more cells were randomly selected from the inDrops without replacement to calculate the simulated spot data.

The results showed that SDePER had robust performance across all cell density settings while GLRM had decreasing performance when the cell density increased (Fig. S6) even when there were no platform effects (internal reference).

The descriptions of the simulation were added in the Method (page 30 line 635-641). The results were added to Results (page 7 line 134-142) and supplementary materials (Fig. S6)

**2. Real datasets have "vague" and "rare" cell types (Fig. 5) in the ST spot data. The claim that the approach can handle tissue samples with "rare cell types" also needs simulation based evaluation. For example, the authors could consider varying the minimum number of cells of a cell-type required to be present (in scRNAseq reference and simulated ST data) for a cell to be correctly deconvolved.**

*Response:* To assess the robustness of SDePER to rare cell types, we conducted the following two simulation analyses by choosing oligodendrocytes (“Oligo”) as the “rare cell type” for investigation.

First, we down sampled Oligo cells in the reference scRNA-seq data for multiple times and used each down sampled reference data to deconvolve the STARmap-bases simulated ST data. The performance of SDePER on Oligo cells was evaluated (Fig. S4) using RMSE, false negative rate (FNR) and false discovery rate (FDR). For external reference, the performance is robust to the number of Oligo cells in the down sampled reference data. For internal reference, when there were more Oligo cells, the median RMSE remained unchanged with shorter interquartile range suggesting a more stable results when there were more Oligo cells.

In the second simulation analyses, we examined the performance of SDePER on Oligo using groups of spots stratified based on the number of Oligo cells per spot from the STARmap-based simulated ST data. All the simulated spots were divided into groups based on the total number of cells (n) and the number of oligodendrocytes per spot. Within each group of spots with the same total number of cells, both the relative absolute error (RAE) and the false negative rate (FNR) decreased when the number of oligodendrocytes per spot increased (Fig. S3). Specifically, when there were at least 3 cells in the spot, the rare cell type could be always identified as present (FNR=0). When there were only 2 cells in the spot, SDePER had over 87% chance to identify the rare cell type as present (FNR=0.125). This trend is consistent across spot groups with different total number of cells and between external and internal reference.

In summary, these results suggested that the performance of SDePER is robust to rare cell types in the reference scRNA-seq data but worse for the rare cell types in the ST data, especially when there are less than 2 cells in the spot.

These results were added as a new subsection titled “Robustness of SDePER to rare cell types” under the Results section (page 9-10 line 177-195) and in the supplementary materials (Fig. S3, S4).

**3. For the real datasets in Fig. 3-4, the authors annotate their spatial data with 4 very broad cell-types (Fig. 3a, Fig. 4a). Could the authors clarify how they annotated the spatial data to obtain the patterns shown in Fig. 3a and Fig. 4a used as ground truth?**

*Response:* We followed the annotation of these datasets from the previous publications. For the MOB data (Fig. 3a), we accommodated the ST data annotation by CARD [5], which was obtained based on the H&E image, overlayed spatial transcriptomics locations on top of the H&E image and manually annotated each measured location in spatial transcriptomics with the tissue structure annotations extracted from the H&E image (page 10 line 204).

For the melanoma data (Fig. 4a), we borrowed the annotation from the original publication of the data by Thrane et al. (2018) [6] and BayesSpace [2]. In Thrane et al. (2018), the H&E image was manually annotated by a trained pathologist to identify melanoma, stromal and lymphoid tissue. Authors of BayesSpace conducted clustering on the spatial data and annotated the unannotated area in the original paper. We took the annotation from both publications for a complete annotation for the slice.

**4. Could the authors include visualizations of the reference scRNA-seq datasets for all of the analyses to improve readability? These can be UMAP visualizations with annotated cell types.**

*Response:* We added UMAPs of the scRNA-seq dataset with cell type annotation in the supplementary material (Fig. S10, Fig. S15, Fig. S19 and Fig. S22).

**5. The pseudo-spot data obtained from scRNA-seq data was used in the model but not used in a supervised learning manner. To show that including such data is beneficial, an ablation test needs to be included to compare results using and not using the pseudo-spot data.**

*Response:* We conducted an ablation test by running SDePER on simulated data from STARmap-based simulation, sequencing-based simulation and the simulations for high cell density with and without pseudo-spot data. The results showed a significant drop in the performance of SDePER when no pseudo-spot data was included in the training, suggesting the importance of including the pseudo-spot in the CVAE training. We added these results in the supplementary material (page 8 line 144-160)

**6. To show the necessity of using the regularization to enforce the two constraints: sparseness of cell types in spots and correlation between neighboring spots, ablation tests are needed. In particular, in Fig. 3 and 4, a small number of cell types are considered. In such cases, will the sparseness constraint still be useful?**

*Response:* We conducted ablation tests on SDePER to evaluate the necessity of the two penalties by comparing the performance of SDePER with and without each penalty. The results showed that both the adaptive LASSO penalty and graph Laplacian penalty had neglectable contribution to the RMSE but did contribute to lower the false discovery rate with the adaptive LASSO penalty having slightly larger contribution. Details on the ablation test were added to the Method (page 30 line 648-657). The results were added to the Results (page 8 line 144-167) and the supplementary material (Fig. S7 and S8).

In addition, we conducted additional simulations for small number of cell types. We reduced the total number of cell types in the STARmap-based simulations to 5 and specifically performed ablation test on SDePER to see if the adaptive LASSO is useful. The results (Fig. S9) showed that the adaptive LASSO penalty had neglectable contribution to lowering the RMSE but did help with the false discovery rate (FDR). The results were added to the Results (page 8 line 150-152) and supplementary materials (Fig. S9).

**7. More details on hyperparameter tuning is needed for users to be able to set hyperparameters when using this tool. In particular, how are hyperparameters for the CVAE model chosen for each dataset? In the paragraph from line 417-422: what is the range of parameter search? How to use simulated data to determine best parameters?**

*Response:* The hyperparameters in SDePER include hyperparameters of the neural network structure in CVAE, the weight for sparsity penalty and graph Laplacian penalty in GLRM, and the Gaussian kernel hyperparameters in imputation.

Hyperparameters of CVAE include the dimension of the latent space, the number of hidden layers in the encoder and decoder, and the number of neurons in each layer. We set the number of neurons in latent space as 3 times the number of cell types in the reference scRNA-seq data to ensure we have ~3 dimensions to represent each cell type. We use 1 hidden layer for both encoder and decoder based on experience. The number of neurons in each hidden layer is the largest integer no more than the geometric mean of the number of neurons in the input layer and latent space to enforce dimension reduction.

We tuned the weights ($\lambda_{r}$ and $\lambda_{l}$) for sparsity penalty and graph Laplacian penalty in GLRM component using 5-fold cross validation The cell type marker genes identified from the transformed reference scRNA-seq data were randomly divided into 5 groups with equal size. Each group was considered as validation data and the rest groups were used as training dataset. For given values of $\lambda_{r}$ and $\lambda_{l}$, the training data was used to fit the GLRM model, which was used to calculate the log likelihood of the validation data using the base model. The log likelihood of the 5 validation datasets was averaged and compared across different settings of $\lambda_{r}$ and $\lambda_{l}$. The setting that achieved the largest average log likelihood was chosen. By default, the range of both hyperparameters was set from 0.1 to 100, and 8 values were evenly selected on a log scale (geometric progression) from this range. Detailed descriptions were added to the Method section (page 24 line 516-522).

For hyperparameters in the Gaussian kernel used in imputation, we determine the optimal values using the STARmap-based simulated data. We conducted coarse-graining procedures on the STARmap data to generate simulated ST dataset with different spot size varying from 100 × 100 to 1000 × 1000 with an interval of 100, which correspond to high to low resolution. In each simulated ST dataset, we know the true cell type proportions in each spot. For a given setting of $\phi$ and $\tau^{2}$, we impute the cell type proportions using the simulated dataset with spot size 1000 × 1000 to reconstruct spatial maps with different resolutions higher than 1000 x 1000 (smaller spot size). Then we compare the imputed cell type proportions to the ground truth and calculated the average RMSE across the different higher resolution levels. The hyperparameter setting that achieved the smallest average RMSE was chosen. The search ranges for $\tau$and $\phi$ are both [1 μm, 200 μm]. Revisions were made in Method (page 26 line 555-564) for clarification this.

**8. For Fig. 2B and 2D, please explain how many data points are there in each box and what each point corresponds to.**

*Response:* Each point is one spot and there are 581 data points (spots) in each box in total. We added the descriptions in the legend of Figure 2.

**Some minor comments:**

**Line 65: Despite the success (remove "of")**

**Line 66: Address"es" them simultaneously**

**Line 70: "and varying"**

**Line 72: "were shown in a previous"**

**Line 96: "removes" is a strong claim that is not sufficiently justified. Consider "corrects for"**

**Line 110: "using external reference" consider adding "an" or "the" 
Consider swapping Fig. S1 and S2 order since S2 is mentioned before S1 in text**

**Line 138: "on real data"**

**Line 143: "composed of one cell type"**

*Response:* We thank the reviewer for the suggestions. We corrected our writings accordingly.

**Fig. 3B-C: Could the authors comment on Spotlight as well since the results look quite poor**

*Response:* SPOTlight suggests to down-sample cells to 100 cells per cell type.
This may introduce randomness and bias to the results (https://marcelosua.github.io/SPOTlight/articles/SPOTlight_kidney.html). This was added to the Results section (page 11 line 211-213).

**Line 219: It is not obvious how Fig. 5F demonstrates by "visualization" and Fig. 5G by "quantitative measure".**

*Response:* For each layer, we identify one region-specific marker gene by comparing spots from the region to the other spots using Seurat. Figure 5F visualizes the average imputed gene expression of the region-specific maker genes at locations inside each layer. A red diagonal indicates that each layer marker gene was imputed to have high expression in the layer that it is the marker for and low expression in the other layers for which it is not a marker for. This suggests that the imputed gene expression is accurate. In Figure 5G, we calculated the ratio of the average imputed expression levels of the layer marker gene in the layer that it’s a maker for to the other layers. Higher ratio showed a more different imputed expression levels of each layer marker genes between its represented layer and other layers. Revisions were made in the legend of Fig. 5F and Fig. 5G for clarifications.

**Line 400: Could the authors explain this criteria? How do they account for biological holes in tissue slices?**

*Response:* The equation in Line 400 defines locations to be imputed in the spatial map. We first use the “finding contour” function in opencv [7] to determine the contours of the tissue edge which can identify outlines of the tissue edge and biological holes inside the tissue. Spots closest to the identified edge of tissue and biological holes are considered as edge spots. All other spots are considered as inner spot.

For us to impute for a given position on the new spatial map with higher resolution, if it is outside the tissue or within a biological hole, it does have any original spot nearby so we do not have enough information to conduct the imputation. We use the equation in Line 400 to make sure we only impute for locations that have are not far from the measure region on the slice. Locations that are far from measured regions can be easily filtered out. Locations inside the measure regions but far from the edge of the tissue or biological holes will satisfy the first criteria in the equation: $\text{min}_{\left\{ i=1,\ldots,N; i is an inner spot \right\}}\left\| c_{i^{*}}-c_{i} \right\|\leq D$. Locations around the edge of the tissue or biological holes would not satisfy the first criteria. So we use the second criteria to filter them: $\text{min}_{\left\{ i=1,\ldots,N; i is an edge spot \right\}}\left\| c_{i^{*}}-c_{i} \right\|\leq\frac{D-d}{2}$. Basically, we intend to only impute for locations within D/2 distance from the edge spot center. We used the threshold of (D-d)/2 because a small square is constructed centered at the location $i^{*}$ and the side length of the square is d. To make sure the whole square is within the D/2 radius from the edge spot center, we need to require the center of the square to be no more than (D-d)/2 away from the edge spot center. Revisions were made in Method section (page 25 line 537-538) for clarifications.

**Fig. 3: Why do the authors choose different cell-type specific genes in Fig 3a and 3e?**

*Response:* Genes in Fig 3a were cell type marker genes for the dominant cell in each layer based on original publication and CARD paper [5]. Genes in Fig. 3e were layer-specific marker genes whose expression level can distinguish each layer from the others. The layer-specific markers were computationally identified by comparing spots across different layers Seurat. These can be found in line 201-206 on page 10.

**Fig. 6C. Incorrect legend. Fig. 6C is not a heatmap.**

*Response:* We changed the legend of Fig. 6C to “The estimated cell-type proportions on each location for SMC, Ciliated cells, AT1 and AT2 cells inferred by SDePER, RCTD, SpatialDWLS, and DestVI”.

**Fig. S3 is the same as 3A markers with the additional EPL-IN population which is not in 3A. Could the authors add this annotation /marker to 3A?**

*Response:* We used Figure 3A to demonstrate that the dominant cell types are representative of the annotated layers and therefore in 3B, we could use the dominant cell type to calculate the similarity between our results and the ground truth. In Figure 3A, there are only 4 annotated layers and each has one dominant cell type. EPL-IN is not the dominant cell type for any layer so we did not show the expression of Kit. The original Fig. S3 (Fig. S10 in this revised manuscript) was used to show the distribution of all cell types, which did indicate that EPL-IN was not dominant in any layer.

Taken together, we believe that adding EPL-IN out in Fig. 3A would cause confusions on the dominant cell type in each layer. Therefore, we kept Fig. 3A and Fig. S3 (Fig. S10 in this revised manuscript) unchanged.

Reference:

1. Rossi, A., et al., *Stromal and Immune Cell Dynamics in Tumor Associated Tertiary Lymphoid Structures and Anti-Tumor Immune Responses.* Front Cell Dev Biol, 2022. **10**: p. 933113.

2. Bergomas, F., et al., *Tertiary intratumor lymphoid tissue in colo-rectal cancer.* Cancers (Basel), 2011. **4**(1): p. 1-10.

3. Sautes-Fridman, C., et al., *Tertiary lymphoid structures in the era of cancer immunotherapy.* Nat Rev Cancer, 2019. **19**(6): p. 307-325.

4. Vannan, A., et al., *Image-based spatial transcriptomics identifies molecular niche dysregulation associated with distal lung remodeling in pulmonary fibrosis.* bioRxiv, 2023.

5. Ma, Y. and X. Zhou, *Spatially informed cell-type deconvolution for spatial transcriptomics.* Nat Biotechnol, 2022. **40**(9): p. 1349-1359.

6. Thrane, K., et al., *Spatially Resolved Transcriptomics Enables Dissection of Genetic Heterogeneity in Stage III Cutaneous Malignant Melanoma.* Cancer Res, 2018. **78**(20): p. 5970-5979.

7. Bradski, G., *The openCV library.* Dr. Dobb's Journal of Software Tools, 2000. **25**(11): p. 120-125.

**Second round of review**

**Reviewer 1**

The authors have addressed most of my concerns. Below are my follow-up comments after reviewing the resubmitted manuscript:

1. Although the computation time has been reduced compared to the initial version (8.58 hours using a 64-core CPU machine on 4992 spots; 0.56 hours using a 20-core CPU machine on 282 spots), it remains significantly longer than that of existing widely used methods benchmarked in this study (or see in https://www.nature.com/articles/s41592-022-01480-9.pdf and https://www.nature.com/articles/s41592-022-01480-9.pdf) and in the previous publications on spatial cell type deconvolution (Celloscope and spatialDWLS) in Genome Biology. For example, Celloscope took only 0.11 hours on a dataset with 274 spots, while spatialDWLS required 23 minutes for a dataset with 2698 spots. If users only have small datasets, the current approach may be acceptable, but the relatively high computation burden raises concerns about its generalizability to larger datasets and its feasibility for broader usage.

2. To address the issue of losing biological signals in the CVAE adjustment, the authors identified cell type marker genes from the transformed reference scRNA-seq data and used them to fit the GLRM model. However, additional details regarding the selection criteria and the number of remaining genes are necessary for this step.

3. In response to the question about why CVAE was chosen to remove batch effects instead of other methods such as Seurat, it should be noted that Seurat can integrate data not only in the latent space but also on expression data, as detailed in Seurat’s documentation (https://satijalab.org/seurat/reference/integratedata). Further clarification on the decision to use CVAE over other methods such as Seurat would be beneficial.

**Reviewer 2**

The authors have addressed my main concerns.

**Reviewer 3**

My concerns have been addressed.

**Authors Response**

**Point-by-point responses to the reviewers’ comments:**

Reviewer #1’s comments:
The authors have addressed most of my concerns. Below are my follow-up comments after reviewing the resubmitted manuscript:

1. Although the computation time has been reduced compared to the initial version (8.58 hours using a 64-core CPU machine on 4992 spots; 0.56 hours using a 20-core CPU machine on 282 spots), it remains significantly longer than that of existing widely used methods benchmarked in this study (or see in https://www.nature.com/articles/s41592-022-01480-9.pdf and https://www.nature.com/articles/s41592-022-01480-9.pdf) and in the previous publications on spatial cell type deconvolution (Celloscope and spatialDWLS) in Genome Biology. For example, Celloscope took only 0.11 hours on a dataset with 274 spots, while spatialDWLS required 23 minutes for a dataset with 2698 spots. If users only have small datasets, the current approach may be acceptable, but the relatively high computation burden raises concerns about its generalizability to larger datasets and its feasibility for broader usage.

Response: We appreciate the comment. We will continue to improve the speed of SDePER to enhance its generalizability to larger datasets by improving the coding and potentially involve GPU computing, which we feel to be out of the scope of this study.

2. To address the issue of losing biological signals in the CVAE adjustment, the authors identified cell type marker genes from the transformed reference scRNA-seq data and used them to fit the GLRM model. However, additional details regarding the selection criteria and the number of remaining genes are necessary for this step.

Response: We added the details of the selection criteria in the first paragraph of the subsection of Graph Laplacian regularized model for cell type deconvolution.

3. In response to the question about why CVAE was chosen to remove batch effects instead of other methods such as Seurat, it should be noted that Seurat can integrate data not only in the latent space but also on expression data, as detailed in Seurat’s documentation (https://satijalab.org/seurat/reference/integratedata). Further clarification on the decision to use CVAE over other methods such as Seurat would be beneficial.

Response: Current batch correction methods strongly rely on the assumption that there are common cell types or shared biological cell states between batches. Specifically, the MNN-based approaches, such as the Seurat Data Integration method, identify pairs of cells from different batches and the difference between cells in each pair is utilized to estimate the batch effects. When scRNA-seq and ST data are considered as two batches by these approaches, each spot from the ST data is a mixture of multiple cells with potentially multiple cell types while each cell from the scRNA-seq data has only one cell type. Unless there are many spots in the ST data that have only one cell type, the identified “cell pairs” do not have the same biological state and the difference between them include both platform effects and cell type composition difference. This causes the estimated batch effects to be larger than the platform effects so these approaches tend to under adjust the data for platform effects.

CVAE is a deep generative model which learns the data distribution in a latent space and a generative process to generate new data points from the learned distribution. It does assume that scRNA-seq data and ST data share the same type of distribution in the embedding space, which is a weaker assumption than the batch correction methods. And we accommodate for this assumption by adding pseudo-spots in the CVAE training data.

To demonstrate the advantage of CVAE, we replaced the CVAE component in SDePER with Seurat Integration method and ran it on the STARmap-based simulated data with external reference. We compared the results to those of SDePER and GLRM (Additional File 1: Fig. S29). The comparison showed that Seurat Integration did correct for certain part of the platform effects so it had certain improvement over GLRM. But SDePER was able to achieve further and larger improvement over Seurat+GLRM, suggesting higher efficiency of CVAE in correcting for platform effects than Seurat.

We added these explanations in the last paragraph of the subsection titled “Conditional variational autoencoder for platform effect adjustment” on page 21-22. The comparison of the results by GLRM, Seurat+GLRM,and SDePER was added to the Supplementary Materials as Additional File 1: Fig. S29.


Reviewer #2: The authors have addressed my main concerns.

Response: We thank the reviewer for the constructive comments.

Reviewer #3: My concerns have been addressed.

Response: We thank the reviewer for the constructive comments.
